# Proneural–mesenchymal hybrid glioblastoma cells are resistant to therapy and dependent on nuclear import

**DOI:** 10.1093/neuonc/noaf160

**Published:** 2025-07-08

**Authors:** Guillaume Bourmeau, Oceane Anezo, Jeremy Raymond, Alberto Ballestín, Cathy Pichol-Thievend, Juliette Reveilles, Adrien Thomas, Lin Wang, Melanie Miranda, Eve Moutaux, Stephane Liva, Valentino Ribecco, Laetitia Besse, Florent Dingli, Damarys Loew, Celine Vallot, Gaetano Gargiulo, Vidhya M Ravi, Kevin Joseph, Giorgio Seano

**Affiliations:** Institut Curie, INSERM U1339—CNRS UMR3666, Tumor Microenvironment Lab, Université Paris-Saclay, Orsay, France; Institut Curie, INSERM U1339—CNRS UMR3666, Tumor Microenvironment Lab, Université Paris-Saclay, Orsay, France; Nuffield Department of Medicine, Ludwig Institute for Cancer Research, University of Oxford, Oxford, UK; Institut Curie, INSERM U1339—CNRS UMR3666, Tumor Microenvironment Lab, Université Paris-Saclay, Orsay, France; Institut Curie, INSERM U1339—CNRS UMR3666, Tumor Microenvironment Lab, Université Paris-Saclay, Orsay, France; Institut Curie, INSERM U1339—CNRS UMR3666, Tumor Microenvironment Lab, Université Paris-Saclay, Orsay, France; Institut Curie, INSERM U1339—CNRS UMR3666, Tumor Microenvironment Lab, Université Paris-Saclay, Orsay, France; Department of Computational and Quantitative Medicine, Hematologic Malignancies Research Institute and Beckman Research Institute, Duarte, California, USA; Single Cell Initiative, Institut Curie, PSL University, Paris, France; Translational Research Department, Institut Curie, PSL University, Paris, France; CNRS UMR3244, Institut Curie, PSL University, Paris, France; Single Cell Initiative, Institut Curie, PSL University, Paris, France; Translational Research Department, Institut Curie, PSL University, Paris, France; CNRS UMR3244, Institut Curie, PSL University, Paris, France; MINES ParisTeach, CBIO-Centre for Computational Biology, PSL Research University, Paris, France; Institut Curie, PSL University, INSERM U900, Paris, France; Institut Curie, INSERM U1339—CNRS UMR3666, Tumor Microenvironment Lab, Université Paris-Saclay, Orsay, France; Institut Curie, Université PSL, CNRS UAR2016, Inserm US43, Université Paris-Saclay, Multimodal Imaging Center, Orsay, France; Institut Curie, PSL Research University, CurieCoreTech Spectrométrie de Masse Protéomique, Paris, France; Institut Curie, PSL Research University, CurieCoreTech Spectrométrie de Masse Protéomique, Paris, France; Single Cell Initiative, Institut Curie, PSL University, Paris, France; Translational Research Department, Institut Curie, PSL University, Paris, France; CNRS UMR3244, Institut Curie, PSL University, Paris, France; Max-Delbrück-Center for Molecular Medicine (MDC) in the Helmholtz Association, Berlin, Germany; 3D-Brain Models Lab for Neurodegenerative Diseases, Medical Centre, University of Freiburg, Freiburg, Germany; Department of Neurosurgery, Medical Center, University of Freiburg, Freiburg, Germany; 3D-Brain Models Lab for Neurodegenerative Diseases, Medical Centre, University of Freiburg, Freiburg, Germany; Department of Neurosurgery, Medical Center, University of Freiburg, Freiburg, Germany; Institut Curie, INSERM U1339—CNRS UMR3666, Tumor Microenvironment Lab, Université Paris-Saclay, Orsay, France

**Keywords:** cell plasticity, glioblastoma, hybrid, nuclear transport, resistance mechanisms

## Abstract

**Background:**

Despite extensive research efforts, glioblastoma (GBM) remains a deadly disease with poor prognosis. Although previous studies have identified various cell states within GBM tumors, the molecular mechanism underlying adaptive GBM cell plasticity induced by conventional therapy remains unclear.

**Methods:**

We used fluorescent reporters for proneural (PN) and mesenchymal (MES) subtypes to monitor GBM cell plasticity in real-time across multiple patient-derived cell lines. This approach revealed cells that concurrently expressed both PN and MES markers. To investigate this unique hybrid population, we implemented a comprehensive methodological approach encompassing bulk and single-cell RNA sequencing, single-cell ChIP sequencing, nuclear proteomics, high-resolution imaging, orthotopic mouse models, clinical dataset analysis, and pharmacological and genetic techniques. This multifaceted strategy allowed us to gain functional and molecular insights into this distinct cellular population.

**Results:**

We showed that these hybrid cells are increased by conventional therapies, and are resistant to these therapies. At the molecular level, hybrid cells display significant alterations in chromatin structure and nuclear protein composition, elevated transcriptional activity, Myc activation, and improved transport between the nucleus and cytoplasm. Genetic and pharmaceutical inhibition of the nuclear import/export shuttling machinery, increased in hybrid cells, effectively suppressed adaptive GBM cell plasticity and hybrid identity, thereby enhancing the sensitivity of GBM cells to therapies.

**Conclusions:**

Our results indicate that GBM hybrid cells play a crucial role in chemoradiation resistance. The nuclear transport machinery presents a potential therapeutic target for hybrid cells, offering a way to counteract the typical resistance to treatment observed in GBM.

## Graphical Abstract

**Graphical Abstract:**
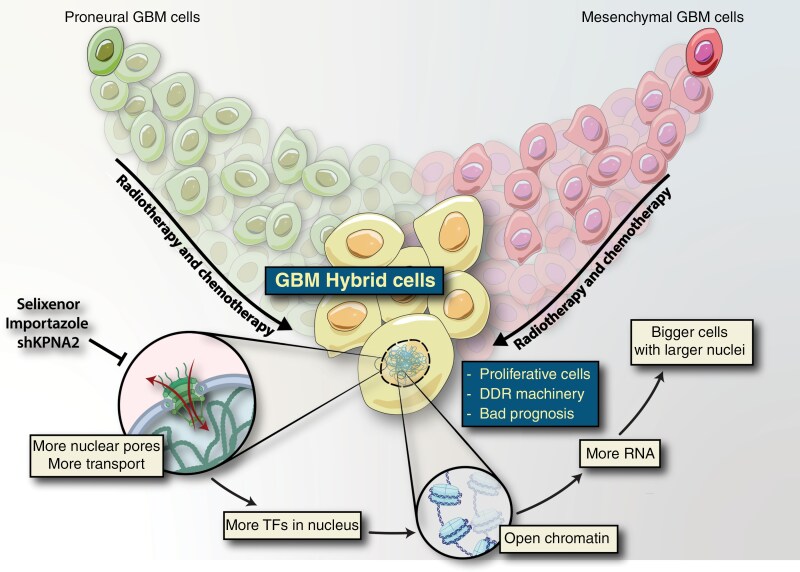

**Key Points**

- Hybrid glioblastoma (GBM) cells are highly proliferative and resistant to therapies.
- Hybrid cells are enriched at recurrence and are a bad prognosis factor for patients.
- Hybrid cells are therapeutically vulnerable to nuclear shuttling machinery targeting.

**Importance of the Study**

Although significant efforts have been made to classify and treat glioblastoma (GBM), it remains a highly lethal disease, with no substantial therapeutic advancements in the past 2 decades. The primary clinical obstacle for patients is therapy resistance, and it has become clear that the current classifications and known cell states are inadequate for identifying the GBM cell population responsible for disease recurrence. In this light, our findings, demonstrating the existence of a resistant GBM hybrid cell population at the interface between the proneural and mesenchymal subtypes, may open a new clinically relevant avenue of research, in which the molecular vulnerabilities of hybrid cells, such as nuclear transport, may represent new potential targets.

Glioblastoma (GBM) is among the most lethal cancers.^1,2^ Standard treatment includes surgery, radiation, and temozolomide (TMZ) chemotherapy.^3^ Despite these treatments, median patient survival is 8 months, with only 6.9% surviving 5 years post-diagnosis due to recurrence.^4^ Over the past 2 decades, clinical trials for various therapies have not significantly improved outcomes.^5,6^

The failure to treat GBM effectively stems from both extrinsic factors, such as low drug exposure due to the blood–brain barrier, and intrinsic factors, specifically GBM cell heterogeneity and plasticity. Bulk transcriptome analyses have shown that GBM tumors can be broadly classified into proneural (PN), classical (CL), and mesenchymal (MES) transcriptomic subtypes, demonstrating intertumor heterogeneity.^7,8^ Using single-cell RNA analysis, Neftel et al.^9^ have revealed oligodendrocyte progenitor cell (OPC)-like, astrocyte (AC)-like, neuronal progenitor cell (NPC)-like, and MES-like transcriptional states within each GBM, underlying intratumor heterogeneity.^9^ Each tumor has a dominant cell state, with MES and classical subtypes enriched in MES-like and AC-like states, and proneural subtype enriched in both OPC-like and NPC-like cell states.^10^ Moreover, longitudinal studies have shown interactions between the microenvironment and phenotype at recurrence.^10,11^

Despite extensive insights into GBM classification,^12–15^ the therapeutic potential of GBM cell plasticity, that is, the potential to transition between states, remains unexplored due to a lack of selective vulnerabilities.

GBM phenotypic plasticity encompasses both defined cell identities, evolving along a single PN to MES axis in vitro, in animal models and patients.^16–20^ However, longitudinal studies of GBM patients showed that the majority of tumors retained the same subtype at recurrence, and subtype switching lacks a preferential directionality.^8,10^ These results suggest that GBM plasticity and therapy resistance are not explained solely by the extremes of this axis of variation. Additionally, the weak correlation between individual cell state and patient prognosis suggests significant involvement of GBM cell plasticity, leading us to hypothesize that resistance and recurrence may be due to intermediate hybrid cells within the PN–MES axis. Despite computational reports predicting the existence of these hybrid cells,^9,17,18^ no published study has characterized them, highlighting a critical knowledge gap in understanding and treating GBM. Thus, we focused on functionally investigating the unique characteristics and vulnerabilities of hybrid GBM cells.

This study utilized previously validated reporters of PN and MES identities to track GBM cell plasticity, revealing a distinct hybrid cell population with a unique transcriptomic, chromatin, and proteomic profile. Hybrid cells, resistant to and enriched by chemo- or radiotherapy, exhibited enhanced nuclear import/export machinery and increased proliferation. Inhibiting the nuclear import/export machinery, which is overrepresented in hybrid cells, hinders the maintenance of hybrid cells, thus sensitizing GBM cells to conventional therapies. These findings offer insights into the intrinsic and adaptive plasticity of GBM cells, suggesting new strategies for effective therapeutic development.

## Methods

For detailed protocols, see Supplementary Information.

### GBM Subtypes Reporters’ Model

**Figure 1.**
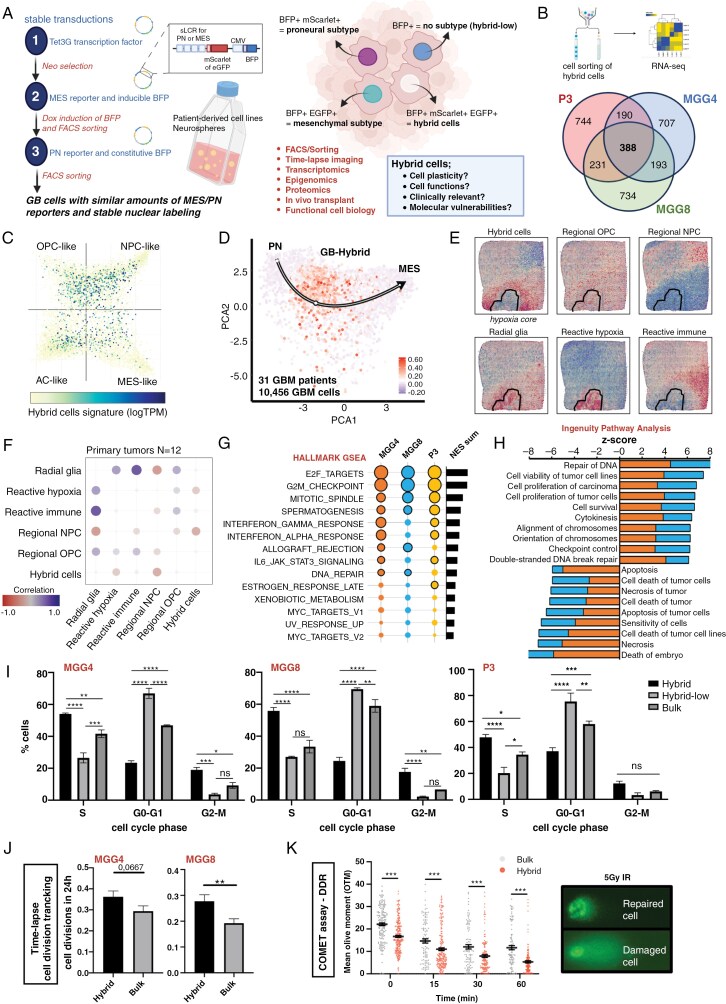
Hybrid GBM cells: a cell proliferative and resistant population with a distinct transcriptomic profile. (A) Schematic of the fluorescent reporter model. eGFP and mScarlet reporters for mesenchymal and proneural GBM signatures, respectively, were sequentially transduced into patient-derived cell lines. Fluorescence levels represent the subtype identity of the cell. Cells also express a constitutive BFP. Schematics created with BioRender.com. (B) RNA sequencing of MGG4, MGG8 and P3 Hybrid cells. Venn graph of the differentially expressed genes in MGG4, MGG8 and P3 Hybrid cells (genes with P-value < .05) compared to hybrid-low cells. (C) Cell-state heterogeneity of hybrid cells, spread over the 4 states described by Neftel et al. performed on https://singlecell.broadinstitute.org/single_cell using hybrid cells signature. From lower expression of the signature in white to high expression in blue. (D) Feature plot for hybrid signature (100 genes) in the proneural–mesenchymal axis. RNA-velocity from Wang et al. (2022)^18^ (31 patients with GBM and 10456 cells in total). (E) Representative surface plot of spatially resolved expression of Hybrid signature and spatial subtypes signatures in patients. Normalized GSEA score is color coded, with red dots being a high expression of the corresponding signature. (F) Correlation plot between hybrid signature and published GBM spatial subtypes in primary GBM patients. (G) Bubble plot of GSEA analysis of MGG4, MGG8 and P3 Hybrid cells for the Hallmark dataset. Size indicates NES, and black borders mean FDR *q-*values < 0.05. Pathways are ranked by the sum of NES in all cell lines, represented by the histograms. (H) Ingenuity Pathway Analysis (IPA) predicted biological functions of MGG4 and MGG8 Hybrid cells. A positive *Z*-score indicates a predicted activated function in Hybrid cells, and a negative *Z*-score an inhibited one. All functions are significant. Orange, MGG4; blue MGG8. (I) Proliferation analysis of sorted Hybrid, bulk and Hybrid-low cells in MGG4, MGG8 and P3 cells using EdU incorporation and a DNA stain. The percentage of cells in S-phase is higher in Hybrid cells in both cell lines. (*n* = 3 for Hybrid and Hybrid-low, *n* = 2 for bulk; ns, nonsignificant; **P < .01; ***P < .001; ****P < .0001; two-way ANOVA). (J) Number of cellular divisions recorded by time-lapse imaging, in Hybrid, bulk and Hybrid-low cells. (*n* = 700 cells examined over 3 independent experiments per condition; ns, nonsignificant; **P < 0.0001; unpaired *t*-test). (K) Time-lapse comet assay of bulk and Hybrid MGG8 cells, showing that Hybrid cells take less damage after irradiation than bulk cells. The olive moment is a quantification of the amount of DNA breaks over total DNA (*n* = 2; ***P < .001; ****P < .0001; two-way ANOVA).

To characterize GBM cell subtypes and monitor changes in real time, we utilized transcriptional synthetic reporters of PN and MES identities, previously validated in vitro and in vivo.^19^ We developed a protocol involving three consecutive lentiviral transductions per cell line to ensure comparable reporter amounts in the nucleus. First, MGG4, MGG8, and P3 cells were infected with pLV[Exp]-CMV > Tet3G(ns):T2A:Neo lentiviral particles. Following neomycin selection, cells were transduced with pLV[Exp]-{MGT#1}>NLS_EGFP:WPRE-TRE3G > SV40-NLS/TagBFP2/cMYC-NLS (MES reporter), driving eGFP expression based on the MES subtype transcriptional expression. Transduced cells were sorted using BD FACSAria^TM^III sorter, with doxycycline-inducible TagBFP2 in the second vector to maintain consistent basal vector expression. Following doxycycline washout, cells were transduced with pLV[Exp]-{PNGT#2}>SV40-NLS/{mScarlet-I}/cMYC-NLS:WPRE-CMV > SV40-NLS/TagBFP2/cMYC-NLS, driving mScarlet-I protein based on the proneural subtype transcriptional signature. Cell sorting was made based on a similar intensity of the constitutive TagBFP2 protein. Post-selection, each cell line expressed constitutive TagBFP2 and two fluorescent proteins reflecting their subtype identity (Figure 1A). We used VectorBuidler Inc. to generate vectors.

### FACS Analysis

#### Hybrid cells, Cell Death, and Size.

Cells were displayed based on FITC and PE-A channels, with the highest fluorescence in both channels gated as hybrid cells. Live single cells were gated for size, and mean FSC-A was recorded. Dead cells were identified using the Alexa-647 channel for far-red SYTOX.

#### Cell Cycle.

10 uM EdU was added for 1 h at 37°C, and incorporation was assessed using the Click-iT EdU Alexa Fluor 647 kit (Thermo Fisher #C10419) following the manufacturer’s instructions.

#### Immunolabelling.

For histone, cells were fixed with 4% PFA, permeabilized using 90% methanol, and incubated with H3K4me3 primary antibody (1:1000; Cell Signaling Technologies, #9751), followed by Alexa Fluor 647 secondary antibody (1:500). For MYC, cells were fixed using 4% PFA, permeabilized with PBS 0.3% Triton X-100, and blocked using PBS, 2% BSA, 0.1% Tween20. Myc primary antibody (1:400; Cell Signaling Technology, 13987) was used, followed by Alexa Fluor 647 secondary antibody (1:500).

### scChIP-seq

Single-cell ChIP-seq was performed as previously described.^21^ Libraries were then sequenced on NovaSeq 6000 in PE100, with a coverage of 100,000 reads per cell (see Supplementary Information).

### Proteomics

MGG8 proteins from cytoplasmic and nuclear fractions were extracted using Qproteome Cell Compartment Kit (Qiagen, 37502). Ten micrograms of proteins were precipitated, trypsinized, and analyzed by LC–MS/MS using an RSLCnano system (Ultimate 3000, Thermo Scientific) coupled with an Orbitrap Eclipse mass spectrometer (Thermo Scientific). Analysis was performed using myProMS^22^ (https://github.com/bioinfo-pf-curie/myproms) (see Supplementary Information)

### Statistical Analysis

Data are mean ± SEM. Two-sided statistical tests were used, with significance set at *P* < .05. Paired or unpaired *t*-tests were applied, and for comparisons involving more than 2 groups, ANOVA with Tukey’s post hoc tests was utilized. Statistical analyses were conducted using Prism software (GraphPad Software Inc.). In vivo experiments and image analyses were conducted blind.

## Results

### Hybrid GBM Cells: A Cell Population With a Distinct Transcriptomic Profile

To study hybrid cells, we transduced patient-derived GBM cell lines cultured as neurospheres with previously published synthetic fluorescent reporters that trace the complex transcriptomic identities of the 2 main GBM subtypes,^19^ MES and PN (Figure 1A, Supplementary File 1). While the reporters underwent previous validation,^19,23^ the examination of cells situated in the middle of the PN–MES continuum remained unexplored. MGG8, MGG4, and P3 cell lines represent different GBM subtypes with distinct mutational and transcriptional backgrounds (Supplementary Figure 1A and B). Engineered GBM cells expressed reporters at varying levels, as indicated by fluorescence intensity. FACS followed by RNA-Seq of the MES and PN subpopulations in all 3 cell lines validated the reliability of subtype reporters, showing enrichment of each subpopulation’s differentially expressed genes in the expected areas of the Neftel et al.’a transcriptome map (Supplementary Figure 1C). Consequently, the double-reporter model proved to be an effective tool for dynamically tracking changes in transcriptomic identity and we identified hybrid cells as characterized by high expression of both PN and MES reporters. We defined the top 10% of GBM cells expressing both MES and PN reporters as hybrid cells, and the lowest 10% as hybrid-low cells (Supplementary Figure 1D).

RNA-Seq on hybrid and hybrid-low cells from MGG8, MGG4, and P3 lines identified the hybrid GBM cell gene signature, regardless of their transcriptomic and mutational profiles (Figure 1B and Supplementary Figure 1E). The 100 most significantly upregulated genes in all 3 lines were selected as the hybrid cell signature (Supplementary File 2).

To test whether the hybrid cell population was positioned between cell states, we projected the hybrid signature onto the cell state map by Neftel et al.^9^ As expected, hybrid cells were widely distributed over the Neftel GBM classifiers and not the extremes of the map, indicating a mixed identity (Figure 1C, Supplementary Figure 1C and F). Additionally, the hybrid geneset showed no significant overlap with the Neftel GBM classifiers (Supplementary Figure 1G). Notably, hybrid cells were located in the middle of the PN–MES axis in a published dataset of over 10 000 GBM cells from 31 patients (Figure 1D) and in the middle of a harmonized scRNA-Seq database with sequencing data from over one million cells from GBM patients^24^ (Supplementary Figure 1H). These findings demonstrated that this double-reporter method efficiently identified an previously uncharacterized hybrid cell population between PN and MES.

Next, we examined the spatial localization of hybrid cells in patient-derived samples. Using the IvyGAP database, which includes RNA-seq data from GBM patients’ tumors at various locations, we discovered that hybrid cells were more prevalent in the non-hypoxic tumor core compared to other regions (Supplementary Figure 2A). Further investigation using spatial transcriptomics on GBM samples from 16 patients revealed that hybrid cells were concentrated in the non-hypoxic core and did not correlate with any previously identified spatial subtypes in primary or recurrent GBMs (Figure 1E-F and Supplementary Figure 2B-C).^25^

These findings showed that hybrid cells occupy a unique, uncharacterized position among multiple GBM classifiers and did not overlap with the existing classification systems.

### Hybrid Cells Exhibit High Proliferation and Enhanced DNA-Damage Repair

To investigate the cellular and molecular properties of hybrid cells, we analyzed enriched pathways from the transcriptome of sorted hybrid cells. Gene set enrichment analysis (GSEA) and over-representation analysis indicated that hybrid cells might exhibit high proliferation and more efficient DNA-damage repair (Figure 1G-H and Supplementary Figure 2D-F and Supplementary File 3).

To validate the transcriptomic enrichment results on proliferation, we conducted EdU-PI cell-cycle analysis and confirmed that hybrid cells were more proliferative than the rest of the cells; over 50% of hybrid cells, compared to 30%–40% of bulk cells, were in the S-phase. Hybrid-low cells were generally less proliferative than bulk cells, indicating that the degree of proliferation followed a gradient of reporter activity (Figure 1I). Notably, not all proliferative cells were hybrid cells (approximately 20% of hybrid-low cells were in S-phase), and not all hybrid cells were proliferative. Using time-lapse microscopy, we confirmed a higher proliferation rate in hybrid cells than in the rest of the GBM cells (Figure 1J).

To validate the enrichment in DNA-damage repair (DDR) pathways (Figure 1G and H, Supplementary Figure 2D–F), we performed a Comet assay. The assay revealed that hybrid cells experienced less damage than bulk cells after 5 Gy of gamma irradiation (IR) at all time points (Figure 1K), indicating hybrid cells were less susceptible to IR-induced DNA damage. Comet assay did not show evident differences in DDR velocity, as has been observed in cancer stem-like cells. Indeed, no differences in the clonogenic capability were detected between hybrid and bulk cells (Supplementary Figure 2G).

Altogether, we discovered that hybrid cells were characterized by high cell proliferation and reduced IR-induced DNA damage.

### Hybrid Cells Have More Transcripts, Larger Size, and Increased Nuclear Shuttling Machinery

**Figure 2.**
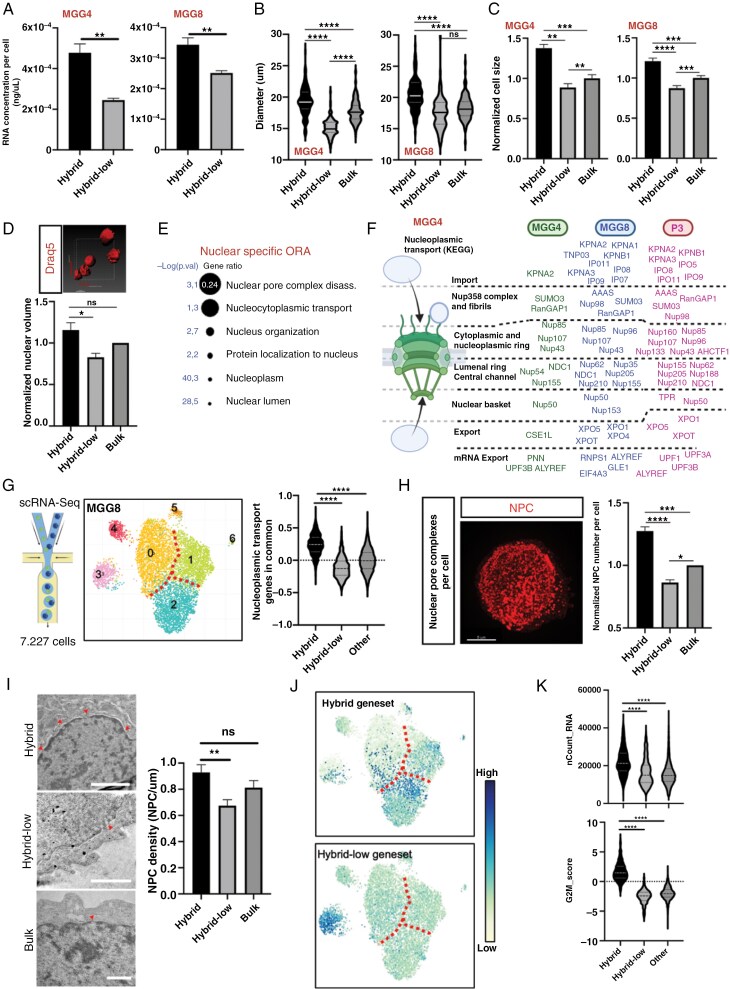
Hybrid cells have more transcripts, larger size and increased nuclear shuttling machinery. (A) Hybrid cells have more RNA per cell. RNA concentrations per cell in Hybrid and Hybrid-low cells in MGG4 (left) and MGG8 (right) (*n* = 4; *P < .05; **P < .01; unpaired *t*-tests). (B) Hybrid cells are bigger. Diameter measurement of Hybrid, bulk, and Hybrid-low cells that adhered on collagen in MGG4 (left) and MGG8 (right) cells (*n* = 300 cells examined over 3 independent experiments per condition; ns, nonsignificant; ****P < .0001; ordinary one-way ANOVA). (C) Normalized cell size of Hybrid, bulk and Hybrid-low cells measured in 3D by FACS, using the FSC measurements. (*n* = 4; **P < .01; ***P < .001; ****P < .0001; one-way ANOVA). (D) Relative nuclei volume of Hybrid, Hybrid-low and bulk MGG8 cells, measured by 3D live imaging (*n* = 2; ns, nonsignificant; *P < .05; ordinary one-way ANOVA). Scale bar (bottom left) 10 uM. (E) Bubble plot of nucleus-related pathways from over-representation analysis querying gProfiler software using the Hybrid signature. Size indicates gene ratio (number of genes of the pathway present in the dataset) and significance is written. (F) Hybrid has upregulated genes in every layer of the shuttling machinery. Upregulated nuclear shuttling genes in MGG4 Hybrid cells (left), MGG8 Hybrid cells (middle), or P3 Hybrid cells (right). Schematics created with BioRender.com. (G) (Left) MGG8 single-cell RNA-seq. UMAP dimensionality reduction resulted in 7 clusters. (Right) Hybrid cells are enriched in nuclear shuttling genes (Common nucleopore genes score) ****P < .0001. (H) High-resolution 3D imaging of NPC (Left) Representative image of NPC imaging. Each dot is a single NPC and each cell is imaged as a 3D stack. Scale bar 5 um. (Right) Normalized number of NPC per cell in MGG8 bulk, Hybrid and Hybrid-low cells (*n* = 3; *P < .05; ****P < .0001; ordinary one-way ANOVA). (I) Transmission electron microscopy of MGG8 cells. (Left) Representative images of nuclear membranes, with arrow showing NPCs. Scale bar 2 uM. (Right) Quantification of the density of NPCs in all populations; Hybrid, bulk, and Hybrid-low cells (at least 20 cells were quantified per population per experiment (*n* = 3); ns, nonsignificant; **P < .01). (J) Cluster 2 is the most highly enriched in Hybrid cells (Hybrid geneset) and cluster 3 the one enriched in Hybrid-low cells. (K) Hybrid cells in MGG8 scRNA-seq are proliferative (G2M_score), and have a higher quantity of RNA per cell (n_Count_RNA). ****P < .0001; one-way ANOVA.

Next, we investigated the hybrid cellular features. First, we quantified RNA in the FACS-sorted cells. Hybrid cells from all cell lines had significantly more RNA per cell than hybrid-low cells. (Figure 2A, Supplementary Figure 3A and B). Moreover, time-lapse imaging showed that hybrid cells were larger than the rest of the cells, with cell diameter and volume confirming they were 20-30% larger (Figure 2B and C, Supplementary Figure 3C and D). High-resolution 3D live imaging also showed that the nuclei of hybrid cells were approximately 15% larger than those of other cells (Figure 2D), consistent with the 2D analysis of fixed cells (Supplementary Figure 3E). These observations strongly suggested that hybrid cells require higher genome expression.

Interestingly, several nucleus-related pathways were significantly enriched in hybrid cells (Figure 2E), particularly nucleoplasmic transport and nuclear pore complex (NPC) pathways, at both bulk (Figure 2E and F) and single-cell levels (Figure 2G, Supplementary Figure 3F). This is intriguing, considering that NPCs are the only path for transport in nuclei, which are enlarged in hybrid cells. High-resolution imaging with a spinning-disk microscope confirmed the transcriptomic data, showing more NPC per cell in hybrid cells (Figure 2H). Ultrastructural analysis further validated these results with significantly higher density of NPCs within the nuclear envelope of hybrid cells compared to hybrid-low cells (Figure 2I).

We then investigated hybrid cells’ heterogeneity via scRNA-seq in MGG8 and MGG4 cells. Unsupervised clustering resulted in seven clusters in both cell lines, with Cluster 2 in MGG8, and Clusters 2 and 4 in MGG4 exhibiting the highest hybrid cell signature expression (Figure 2G, Supplementary Figure 3G and H). Notably, cells with the most pronounced hybrid cell signature were enriched in G2M, had more RNA per cell, had higher expression of nucleoplasmic transport genes, and, as expected, were excluded from the hybrid-low cells (Figure 2G, J, K, Supplementary Figure 3F, I, J).

Intrigued by the scRNA-Seq data, we explored a harmonized scRNA-Seq database with over 1 million cells from GBM patients.^24^ Hybrid cells were spread over all cell states, with a slightly higher percentage of proliferative AC-like and proliferative NPC-like cells (Supplementary Figure 4A), indicating their hybrid and proliferative nature. Interestingly, hybrid cells were prevalent in the recurrent tumors (Supplementary Figure 4B). Further analysis confirmed a strong correlation between the hybrid score and the G2M score, enrichment of DDR, nuclear transport, and rRNA transcription in hybrid cells (Supplementary Figure 4C and D). We notably detected a marked methylation of Histone 3K4 in hybrid cells (Supplementary Figure 4D).

These results showed that hybrid cells had more RNA, larger size, more NPCs, and enhanced nucleocytoplasmic transport.

### Hybrid Cells Exhibit Increased Chromatin Accessibility and Active Histone Modifications at the Promoters of Myc Target Genes

**Figure 3.**
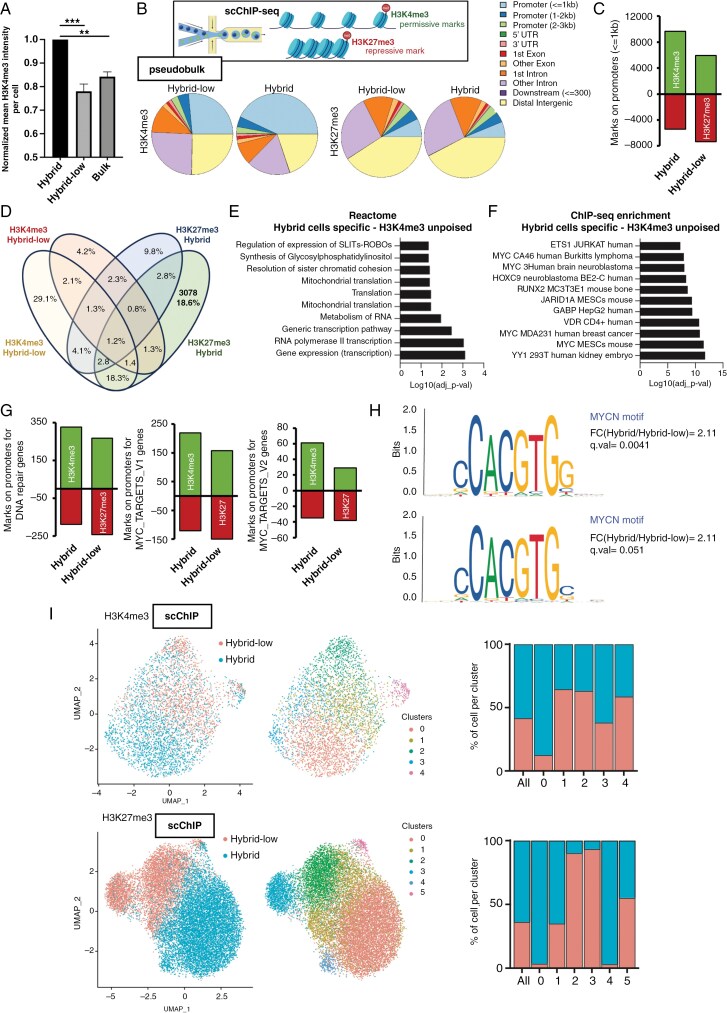
Hybrid cells exhibit increased chromatin accessibility and active histone modifications at the promoters of Myc target genes. (A) Normalized H3K4me3 intensity per cell in Hybrid, bulk and Hybrid-low cells, measured by cytometry (*n* = 3; **P < .01; ***P < .001; one-way ANOVA). (B) (Top) Schematic representation of the single-cell chromatin immunoprecipitation (scChIP) performed. H3K4me3, an active transcription mark; and H3K27me3 a repressive transcription mark, have been immunoprecipitated for sequencing, at a single-cell resolution in MGG8 cells. (Bottom) Pie charts of the annotated H3K4me3 and H3K27me3 peaks in Hybrid and Hybrid-low cells, at a pseudobulk level. H3K4me3 marks are enriched in promoter regions in Hybrid cells, while H3K27me3 does no show differences of localization. Schematics created with BioRender.com. (C) Quantification of the number of H3K4me3 (green) and H3K27me3 (red) marks on proximal promoter regions (<1 kb). (D) Multiset Venn diagram of H3K4me3 and H3K27me3 showing the relatively small overlap of the marked regions in Hybrid and Hybrid-low cells. (E) Reactome enrichment analysis of H3K4me3-specific regions in Hybrid cells, performed using EnrichR, and showing a strong enrichment in pathways linked to transcription. (F) ChIP-seq databases enrichment analysis of H3K4me3-specific regions in Hybrid cells, performed using EnrichR and showing on over-representation of MYC-related datasets. (G) Quantification of the number of H3K4me3 (green) and H3K27me3 (red) marks on (left) DNA repair genes, (middle) Myc_V1 targets genes and (right) Myc_V2 targets genes, all showing an increased number of H3K4me3 in Hybrid compared to Hybrid-low cells. (H) Transcription factor motif analysis of Hybrid H3K4me3 marks, showing an enrichment of MYC/MYCN motifs in these regions. (I) UMAP dimensionality reduction plot of scChIP-seq for H3K4me3 (top) and H3K27me3 (bottom) in MGG8 Hybrid and Hybrid-low cells. In both cases, cell populations cluster separately, with on the right the percentage of cells from each population (Hybrid, blue, and Hybrid-low, red) in each cluster.

Previous studies revealed a spatial link between NPCs and open chromatin,^26^ which prompted us to investigate the chromatin state of hybrid cells. We used FACS to measure H3K4me3, a transcription-activating histone mark, and found higher levels in hybrid cells compared to the other cells (Figure 3A). Using single-cell chromatin immunoprecipitation sequencing (scChIP-Seq) for H3K4me3 and the transcription-inhibitory histone mark H3K27me3,^21^ we observed that hybrid cells had a much higher proportion of H3K4me3 marks at promoters (≤1 kb) than hybrid-low cells, while H3K27me3 regions were similar between the populations (Figure 3B). However, hybrid cells showed more H3K4me3 marks on promoters, whereas hybrid-low cells had more H3K27me3 marks on promoters (Figure 3C). Additionally, hybrid cells displayed more poised chromatin regions (with both H3K4me3 and H3K27me3 marks), suggesting higher plasticity^27^ (Supplementary File 4).

To understand the molecular functions of activated chromatin regions, we analyzed the gene ontology of regions marked exclusively with H3K4me3 in hybrid cells (Figure 3D), and found that these more accessible chromatin regions in hybrid cells were involved in transcription and translation (Figure 3E) and Myc activation (Figure 3F). Promoter regions of genes linked to DDR and of Myc targets showed higher H3K4me3 marks and lower H3K27me3 marks in hybrid cells (Figure 3G). Thus, both transcriptional activation and inhibition play a role in the induction of these pathways in hybrid cells. ChIP-seq for H3K4me3 showed increased marks in DDR gene promoters (ATR, PARP1, and USP39) and Myc target genes (C1QBP, CCT2, CYP4V2, FBLL1, FBLL5, IARS1, KPNB1, LDHA, MAP3K6, and NOLC1) in hybrid cells (Supplementary Figure 5A–C). Consistent with these results, the Myc-specific E-box DNA motif was enriched in H3K4me3 ChIP-IP in hybrid-specific activated genes (Figure 3H).

This scChIP-Seq dataset enabled examination of chromatin heterogeneity and plasticity in hybrid cells at the single-cell level. Unbiased clustering of H3K4me3 and H3K27me3 scChIP-Seq showed that some cells had chromatin features between both sorted populations (CL#1 and #2 for H3K4me3 and CL#1 for H3K27me3), indicating cell plasticity in hybrid cells (Figure 3I).

These results on cell plasticity prompted us to quantify transitions of hybrid or hybrid-low cells over time. Despite the reporters not being time resolute, FACS analysis indicated that both cell types have the potential for transition (Supplementary Figure 5D).

Taken together, these findings suggested that hybrid cells exhibited more accessible chromatin, particularly in relation to Myc target genes and DDR genes, as evidenced by the increased presence of the H3K4me3 mark on promoters.

### Hybrid Cells Have More Proteins Recruited in the Nucleus and Myc Activation

**Figure 4.**
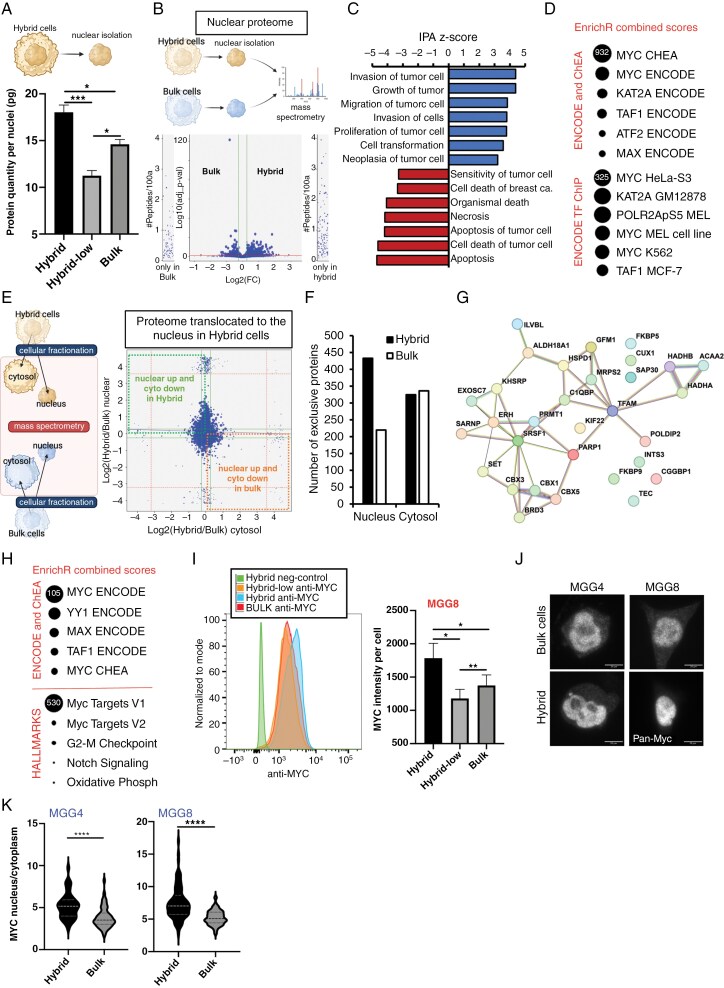
Hybrid cells have more proteins recruited in the nucleus and Myc activation. (A) MGG8 Hybrid cells have more protein per nuclei (*n* = 3; *P < .05; **P < .01; ***P < .001; one-way ANOVA). (B) Volcano plot showing differentially enriched nuclear proteins (FC > 1.2 and *P*-value <.05) between Hybrid and bulk cells. 475 proteins up in hybrid nucleus, 339 in Bulk. (C) Ingenuity Pathway analysis (IPA) predicted biological functions of enriched proteins in Hybrid nuclear proteome compared to bulk. A positive *Z*-score indicates a predicted activated function in Hybrid cells, and a negative *Z*-score an inhibited one. (D) Bubble plot of transcription factor prediction analysis of enriched proteins in Hybrid nuclear proteome compared to bulk. Performed using EnrichR, showing a strong predictive role of Myc. Size indicates EnrichR combined score. (E) (Left) Schematic of the experimental plan, where cytoplasm and nuclear fractions of Hybrid and Hybrid-low cells have been isolated and subjected successively to mass spectrometry analysis. (Right) Double correlation graph of Hybrid/bulk cytoplasmic and nuclear ratios of proteins. Proteins enriched in nuclear and decreased in cytoplasm fractions in Hybrid are in the green dotted square, the ones of bulk being in the orange dotted square. (F) Histogram showing the number of proteins exclusively found in the nucleus or the cytoplasm of Hybrid or bulk cells when compared to each other. (G) STRING protein-protein interaction network of the proteins significantly translocated in the nuclei of Hybrid cells compared to bulk cells. (H) Bubble plot of transcription factor prediction (top) and hallmark pathways enrichment analysis (bottom) of the translocated proteins in Hybrid cells performed with EnrichR, showing an over-representation of Myc. Size indicates EnrichR combined score. (I) Whole cell MYC intensity measured by cytometry in MGG8. (Left) Stacked histogram of Hybrid, bulk and Hybrid-low MYC intensity of a representative experiment. (Right) Normalized MYC intensity per cell in MGG8 Hybrid, bulk and Hybrid-low (*n* = 4; *P < .05; **P < .01; one-way ANOVA). (J) MYC imaging. Representative images of MYC in bulk and Hybrid cells in MGG4 and MGG8. Scale bar 20 um. (K) Myc intensity quantifications presented as ratios between nuclei and cytoplasm of Hybrid cells compared to bulk cells, in both cell lines. (at least 100 cells per condition were examined over 3 independent experiments per condition; ****P < .0001). Schematics created with BioRender.com.

Given that hybrid cells had increased nucleocytoplasmic machinery and open chromatin, we quantified their nuclear proteins. We found that hybrid cells had significantly more proteins in the nucleus than the other cells (Figure 4A). Nuclear proteome (Figure 4B, Supplementary Figure 6A, Supplementary File 5) unveiled higher nuclear concentrations of both PN-like (CBX1, UCHL1, and CRMP1) and MES-like (ANXA1, S100A4, and SARS2) markers in hybrid cells than in the rest of the cells (Supplementary Figure 6B). Enrichment analysis of the nuclear differentially enriched proteins (DEPs, 475 proteins for hybrid, 339 for bulk populations) in hybrid compared to bulk cells revealed activation of proliferation-related functions and inhibition of cell death functions (Figure 4C), corroborating the transcriptional and functional data of hybrid cells (Figure 1G–J). Interestingly, analysis of potential transcription factors (TFs) for nuclear DEPs in hybrid cells identified Myc as a key transcriptional regulator (Figure 4D), aligning with scChIP-Seq results (Figure 3F).

Because of the increased open chromatin and NPCs observed in hybrid cells, we investigated whether enhanced nuclear import/export machinery transports specific proteins from the cytosol to the nucleus by analyzing the nuclear and cytosolic proteomes of hybrid and bulk cells (Figure 4E, Supplementary Figure 6C, Supplementary File 5). The cytosolic proteome showed marked enrichment of cytosolic proteins, validating our methodological pipeline (Supplementary Figure 6D). Hybrid cells had a higher number of unique nuclear proteins than bulk cells (Figure 4F), suggesting activation of specific pathways. To identify proteins differentially translocated to the nucleus in hybrid cells, we selected those with a significant increase in the nucleus and a corresponding decrease in the cytosol. The resulting 31 proteins (Figure 4G, Supplementary File 5) involved G2M checkpoints (KIF22, SRSF1, and SAP30), oxidative phosphorylation (HADH-A and B), methylosome (ERH and PRMT1), Myc and E2F targets, and HP1 complex components (CBX1,3,5) (Figure 4G and H).

Both the proteome and epigenome indicated Myc activation as crucial in hybrid cells; thus, we assessed Myc levels and activation. FACS analysis revealed higher Myc protein levels in hybrid cells compared to bulk and hybrid-low cells (Figure 4I, Supplementary Figure 6E and F) and immunofluorescence confirmed nuclear localization of Myc in both cell lines (Figure 4J–K), verifying Myc activation. Consistently, TCGA, MGG8 scRNA-Seq, and harmonized scRNA-Seq data from GBM patients revealed a strong correlation between MYC or MYCN and the hybrid gene set (Supplementary Figure 6G–I).

These findings suggest hybrid cells have a specific nuclear proteome, particularly with TFs like Myc being actively recruited into the nuclei.

### Hybrid Cells Are Enriched Post-Therapy and Correlate With Poorer Prognosis

**Figure 5.**
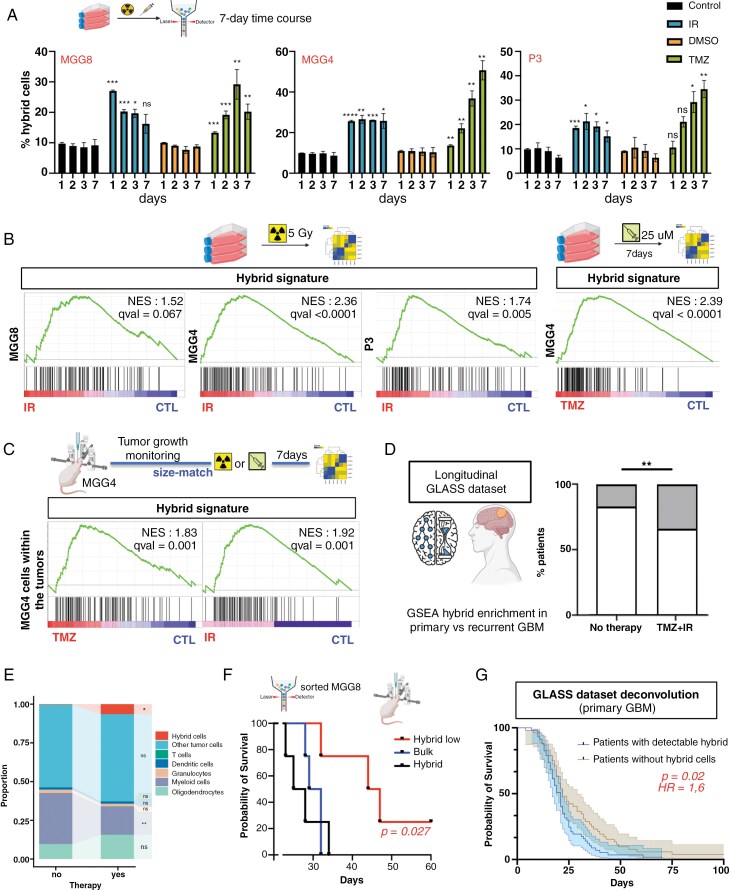
Hybrid cells are enriched post-therapy and correlate with poorer prognosis. (A) Enrichment of Hybrid population under IR treatment (5 Gy) or TMZ treatment (25 uM for MGG4, MGG8, and 200 uM for P3) analyzed from day 1 to day 7 post-treatment by cytometry. (*n* = 3; ns, nonsignificant; *P < .05; **P < .01; ***P < .001; ****P < .0001; multiple *t*-tests). (B) GSEA plot of the Hybrid cells signature in MGG8, MGG4, and P3 cell lines 5 days after 5Gy irradiation and in MGG4 cell line 3 days after 25 uM TMZ treatment. Normalized enrichment score (NES) and FDR *q*-value are indicated. (C) GSEA plot of the Hybrid signature in MGG4-Gluc-GFP tumors orthotopically injected in mice. After tumor growth followed by Gluc, mice were irradiated (10 Gy) or treated with TMZ (10 mg/kg) and tumors were extracted and sequenced 7 days later. Normalized enrichment score (NES) and FDR *q*-value are indicated. (D) Enrichment analysis in paired GBM patient’ tissues from the longitudinal GLASS consortium dataset of the Hybrid signature (IDH-wt glioblastoma only). GSEA was performed on paired tissues (after first vs second surgeries) for each patient. Based on treatments received, patients were stratified in two classes: with TMZ + IR after the first surgery (77 patients), or with no therapy (6 patients). Enrichment was considered when qval was inferior to 0.25. (E) Deconvolution analyses of IDH-wt GBM tumors from the GLASS dataset, showing the average cell state composition of recurrent tumors depending on if chemoradiation was received or not before recurrence. Wilcoxon rank-sum test between primary and recurrent cell proportions performed. ns, nonsignificant; *P < .05; **P < .01. (F) Kaplan–Meier survival curves of mice injected orthotopically with bulk, Hybrid or Hybrid-low MGG8 cells. Log-rank Mantel–Cox test was used for comparison. *N* = 4 mice per group. (G) Kaplan–Meier survival curves of patients based on the presence of Hybrid cells in primary tumors or not after deconvolution. Log-rank Mantel–Cox test was used for comparison. *N* = 109 IDH-wt GBM patients from the GLASS dataset.

Next, we investigated the kinetics of hybrid cells in response to IR or TMZ treatment, standard of care for GBM. MGG4, MGG8, and P3 cell lines were exposed to sublethal doses: 5 Gy IR for all lines or 25 mM TMZ for MGMT-methylated MGG4 and MGG8, and 200 mM TMZ for MGMT-unmethylated P3. Three days post-therapy, hybrid cell proportion doubled with IR treatment, while TMZ resulted in a 3- to 4-fold increase (Figure 5A). GSEA confirmed that the hybrid signature was strongly enriched in all cell lines following IR and TMZ (Figure 5B).

We then studied orthotopic MGG4 tumors treated with IR or TMZ. Using bloodstream GLuc-mediated follow-up, mice with similar tumor burdens were treated with 10 Gy whole-brain IR or 10 mg/kg i.p. TMZ as previously described.^28^ GSEA revealed strong enrichment of the hybrid signature in post-therapy tumors (Figure 5C).

To investigate if conventional therapy-induced hybrid signature enrichment in patients’ GBM, we analyzed the Glioma Longitudinal AnalySiS (GLASS) dataset, focusing on isocitrate dehydrogenase wild-type (IDH-wt) GBM cases. No strong correlations were observed between the proportion of hybrid cells in primary tumors and mutations or copy number variations, suggesting that genomic status does not drive these cells (Supplementary Figure 7A). Interestingly, enrichment of the hybrid signature was observed in recurrent tumors from 30% of the patients who received IR or TMZ between the 2 surgeries, but in only 1 recurrent tumors from 6 patients who did not receive IR and TMZ between surgeries (Figure 5D). Deconvolution analysis of bulk RNA-seq data confirmed hybrid cells enrichment only in recurrent tumors from patients who received chemoradiation between surgeries (Figure 5E, Supplementary Figure 7B).

We then investigated a large transcriptome dataset of 19 GBM patient-derived cell lines treated in vitro with 100 mM of TMZ (the concentration found in the cerebrospinal fluid).^29^ Post-treatment, the hybrid signature was more enriched in non-responders (average viability post-treatment = 90%) compared to responders (average viability post-treatment = 45%) (Supplementary Figure 7C–E). The study indicated that GBM patients with non-responder tumor cells had a worse prognosis than those with responder tumor cells.^29^ Taken together, these findings suggest that hybrid cells contribute to therapy resistance.

Finally, we investigated the aggressiveness of hybrid cells in a preclinical model by orthotopically implanting MGG8 bulk, hybrid-low, or hybrid cells. Mice injected with hybrid cells reached ethical endpoints significantly faster than those with hybrid-low cells but not significantly faster than those with bulk cells, despite a trend (Figure 5F). This survival study indicated higher aggressiveness of hybrid cells. To assess the prognostic impact in patients, we divided IDH-wt GBM patients in the GLASS dataset into 2 groups based on hybrid cells present in primary tumors. GBM patients with detectable hybrid cells had significantly shorter survival, associated with the hybrid population with poor prognosis (Hazard Ratio = 1.6) (Figure 5G).

Our findings indicate that IR and TMZ promote cell plasticity toward a hybrid cell population, highlight the aggressiveness and resistance of this proliferating population, and suggest that therapy-induced hybrid cells contribute to treatment resistance.

### Targeting Nuclear Import/Export Machinery Decreases the Hybrid GBM Cell Population and Enhances Therapy

To target hybrid cells and overcome GBM resistance to chemoradiation, we explored their molecular vulnerabilities. Given the enrichment of nuclear import/export machinery in hybrid cells, we examined it as a potential vulnerability.

**Figure 6.**
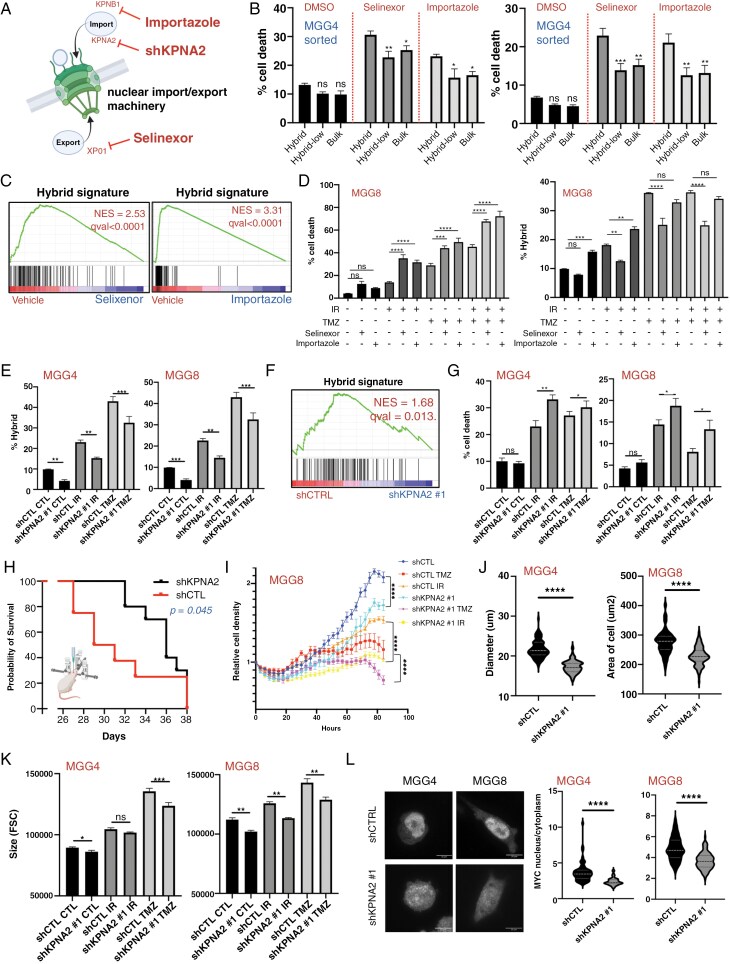
Targeting nuclear import/export machinery decreases the hybrid GBM cell population and enhances therapy. (A) Schematic of the nuclear shuttling machinery targets of the pharmacological inhibitors (Selinexor, importazole) and genetic inhibition (short hairpin against KPNA2). Schematics created with BioRender.com. (B) Cell death of bulk, Hybrid-low and Hybrid cells in MGG8 (left) and MGG4 (right) after treatments with nuclear shuttling machinery inhibitors (3 days, Selinexor 100 nM and Importazole 9 uM) (*n* = 4; ns, nonsignificant; **P < .01; ***P < .001; ****P < .0001; two-way ANOVA). (C) GSEA plot of the Hybrid signature in MGG4 after treatment with Selinexor or Importazole (3 days, Selinexor 100 nM and Importazole 9 uM). (D) Combination of nuclear shuttling inhibitors with conventional therapies increases cell death (MGG8, left) and reduces Hybrid population (right). IR 5Gy; TMZ 10 uM; Selinexor 100nM; Importazole 9 uM (*n* = 5; ns, nonsignificant; **P < .01; ***P < .001; ****P < .0001; one-way ANOVA) (E) Cytometry analysis of the Hybrid cells proportion in control and shKPNA2 MGG4 (left) and MGG8 (right) cells basally and after treatments (5 Gy IR or 25 uM TMZ for MGG4, 5 uM for MGG8) (*n* = 4; ns, nonsignificant; *P < .05; **P < .01; ***P < .001; ****P < .0001; paired *t*-test). (F) GSEA plot of the Hybrid signature in MGG4 after knockdown of KPNA2. (G) Cell death in MGG4 (left) and MGG8 (right) shKPNA2 is increased after therapies with in KPNA2 knockdown cells (*n* = 5; ns, nonsignificant; *P < .05; **P < .01; paired *t*-test). (H) Kaplan–Meier survival curves of mice injected orthotopically with KPNA2 knockdown or control MGG8 cells. Gehan–Breslow–Wilcoxon test was used for comparison. *N* = 8–10 mice per group. (I) Representative growth curves of MGG8 control and shKPNA2 cells over time under after conventional therapies (IR 5Gy, TMZ 10 uM), using incucyte live-cell imaging system (*n* = 3; ****P < .0001; one-way ANOVA). (J) Cell size of shKPNA2 cells compared to a control short hairpin in 2D by imaging, quantified as diameter for MGG4 and cell area for MGG8. (at least 100 cells per condition were examined over 3 independent experiments; ns, nonsignificant; ****P < .0001; unpaired *t*-test). (K) 3D Cell size of MGG4 and MGG8 shKPNA2 cells compared to their respective controls, with or without therapies (IR 5Gy, TMZ 10 uM MGG8, TMZ 25 uM MGG4) using the FSC measurement in cytometry (*n* = 4; ns, nonsignificant; *P < .05; **P < .01; ***P < .001; paired *t*-tests). (L) MYC subcellular quantification in shKPNA2 cells by imaging. (Left) Representative images of MYC in shCTL and shKPNA2 cells in MGG4 and MGG8. Scale bar 20 uM. (Right) Myc intensity quantifications presented as ratios between nuclei and cytoplasm of shCTL compared to shKPNA2, in both cell lines (*n* = 150 cells examined over 3 independent experiments per condition; ***P < .001; ****P < .0001).

First, we tested a pharmacological approach to inhibit the nucleoplasmatic transport machinery (Figure 6A) using importazole (a small molecule that inhibits importin-beta and nuclear import) and selinexor (a clinical drug that targets exporting and nuclear export). Both induced higher cell death in hybrid cells than in the other cells (Figure 6B) and inhibited cell proliferation after 3 days of treatment (Supplementary Figure 8A–C). Transcriptomic analysis showed that the hybrid signature was highly downregulated by both treatments (Figure 6C, Supplementary Figure 8D). Additionally, both drugs inhibited the IR- and TMZ-induced increase in hybrid cells and enhanced IR- and TMZ-induced cell death, with some differences among cell lines (Figure 6D, Supplementary Figure 8E and F), indicating their potential as combinatorial agents with conventional therapies.

Next, we identified and tested a proof-of-concept genetic target for nuclear transport machinery inhibition in hybrid cells. Among the upregulated NPC components in hybrid cells (Figure 2F), importin KPNA2 was highly overexpressed across the 3 cell lines (Figure 6A) and served as a very specific marker for hybrid cells (Supplementary Figure 9A and B).

Thus, we knocked down KPNA2 using multiple constitutive and validated shRNAs (Supplementary Figure 9C) and found that the percentages of basal and therapy-induced hybrid cells decreased, indicating KPNA2’s role in the maintenance or transition to hybrid cells, as shown by FACS (Figure 6E, Supplementary Figure 9D). To confirm the specific decrease in hybrid cells, we also performed RNA-seq, which showed that KPNA2 knockdown decreased the hybrid gene set (Figure 6F). As expected, KPNA2 knockdown increased IR- and TMZ-induced cell death (Figure 6G, Supplementary Figure 9E) and reduced proliferation (Supplementary Figure 9F and G). Testing the functional relevance of hybrid cells in an orthotopic in vivo environment, we found that silencing KPNA2 improved mouse survival (Figure 6H). Time-lapse imaging of KPNA2 knocked down GBM cells exposed to IR or TMZ confirmed that targeting hybrid cells increased the inhibition of proliferation resulting from these treatments (Figure 6I, Supplementary Figure 9H and I). Moreover, cell size, a characteristic of hybrid cells, decreased with KPNA2 knockdown, as observed with selinexor and importazole (Figure 6J and K, Supplementary Figures 8G and 10A).

In line with the hybrid phenotype, DepMap analysis of 52 GBM cell lines showed a strong correlation between KPNA2 expression and Myc_targets_v1 geneset ssGSEA enrichment (Supplementary Figure 10B). Therefore, we tested whether hybrid cells inhibition via shKPNA2 reduced Myc activation. Immunofluorescence showed a clear reduction in nuclear recruitment of Myc (Figure 6L, Supplementary Figure 10C), confirming that Myc activation in hybrid cells is mediated by nuclear shuttling machinery.

These results suggest that the nuclear shuttling machinery is a targetable vulnerability in hybrid cells; hence that targeting nucleocytoplasmic transport could be an effective therapeutic strategy.

## Discussion

This study identifies a GBM cell population at the PN and MES subtype interface, termed hybrid cells. Using a multiomics approach, we found that these cells have markers of both subtypes, greater resistance to therapies, reduced DNA damage, enhanced proliferative activity, more total RNA, larger size, distinct chromatin status and nuclear proteome compared to other GBM cells. Analysis of longitudinal GBM patient and preclinical datasets revealed that this population is enriched at recurrence and is associated with poor prognosis. Mechanistically, the properties of hybrid cells are due to increased nucleocytoplasmic shuttling, which can be pharmacologically targeted, suggesting a new combination therapy to reduce GBM cells’ adaptive plasticity and improve patient survival (Supplementary Figure 10D).

The intrinsic and adaptive plasticity of GBM cells across states remains poorly understood. Recent studies showed that adaptive transcriptional plasticity under therapy is not unidirectional in GBM,^8,18^ and patients mostly do not exhibit subtype switching during progression and recurrence.^8,18,30^ This emphasizes the gap in linking classification with mechanistic insights. Here, we have identified an intermediate hybrid population between known cell states potentially contributing to therapy resistance and recurrence. During cell state changes, transcriptomic data reflect a continuum of gene expression with unclear consequences on cell functions. Thus, there is a need to integrate cell function, organization, and microenvironment into molecular data to define holistic cell states.^31^ Recent technological advancements, like synthetic fluorescent reporters, have enabled us to bridge the gap between transcriptome and phenotype by capturing transcriptomic identities.^23,32^ This approach allowed us to isolate hybrid cells and characterize their properties.

Hybrid GBM cells show upregulation of DDR pathways, including homologous recombination (HR), base excision repair (BER), and mismatch repair (MMR), with increased nuclear levels of DDR proteins BRCA1 and PARP1, key effectors of BER.^33^ This may contribute to hybrid cells’ resistance to TMZ, an alkylating agent causing DNA-damage repaired through BER or MMR,^34^ or IR, which causes DNA-damage repaired by HR.^35^

The high proliferation rate in hybrid cells may also partially explain their DDR. Indeed, open chromatin in actively proliferating cells increases their detection of DNA damage and activation of DDR pathways.^36^ Furthermore, the abundant presence of H3K4me3 in hybrid cells, directly linked to DDR machinery, may contribute to their resistance to TMZ and IR. The nucleotide excision repair mechanism, an alternative pathway for repairing TMZ-induced damage, is also activated during proliferation and relies on PCNA.^37–39^ This may explain why previous studies suggested that proliferation is necessary for DDR.^40,41^ Notably, a recent study showed that TMZ-treated GBM cells resist to therapy with higher DDR by upregulating RRM2, a protein regulating deoxynucleoside triphosphates (dNTP).^42^ Interestingly, we observed increased RNA and protein levels of RRM2 in hybrid cells.

The concurrence of hybrid state and proliferation suggests that the fate of daughter cancer cells might be determined during the active cell cycle. It has indeed been proposed that when these cells enter the cell-cycle, transcriptional programs are reactivated, potentially playing a crucial role in their regulation.^43^ However, answering this question would require more advanced reporters than those currently available.

Previous studies predicted an intermediate state with “cell-cycle properties” that might reactivate at recurrence.^17,18^ Using validated PN and MES reporters, we traced responses driving GBM cells along the PN–MES continuum. Our identification of a therapy-enriched hybrid intermediate population in vitro, in vivo, and in GBM patients, with proliferation and bivalent cell identity, may enable cells to transition and resist therapeutic stress. With upregulated DDR genes and reduced therapy-induced DNA damage, the hybrid cells’ plasticity, marked by nuclear TFs and open chromatin, confers functional advantages under therapeutic stress, making them resilient. These hybrid cells revealed GBM plastic cells’ properties: enhanced transcription, nuclear TFs, open chromatin, increased nuclear pores per cell, and efficient nucleoplasmic transport.

Our findings indicate that GBM cell plasticity is regulated by the nuclear shuttling machinery, which dynamically controls nuclear proteome and epigenome remodeling. Upon activation, Myc transporters to the nucleus, causing hypertranscription, open chromatin^44–47^ and the import of diverse TFs, leading to a phenotype adaptable to environmental changes like the tumor microenvironment or therapeutic stress. Further studies are needed to determine whether hybrid cell plasticity might be influenced by other variables, such as microenvironmental factors,^48,49^ immune response, or paracrine interplay with other GBM cell states.

Inhibiting the shuttling machinery through pharmacological (selinexor, importazole) or genetic (shKPNA2) means decreasing the hybrid phenotype, increasing therapy-induced GBM cell death. This demonstrates the nuclear shuttling machinery’s role in substaining the hybrid phenotype and contributing to GBM aggressiveness. Following encouraging results from a phase II trial of selinexor in patients with GBM,^50^ there is an ongoing clinical trial testing the combination of selinexor with TMZ. While our in vivo studies provided insights into inhibiting hybrid cells via shuttling machinery targeting, we acknowledge limitations in scope and the need for future research expansion. Our investigations used specific preclinical models with constraints like sample size, requiring validation in broader models and larger cohorts to enhance finding generalizability.

Our multiome analyses showed the upregulation of Myc target genes in hybrid cells. We demonstrated that hybrid cells have more Myc in the nucleus and that KPNA2 knockdown reduces the nuclear level of Myc. Thus, enhanced nuclear shuttling machinery in hybrid cells leads to nuclear import of Myc and other TFs, leading to the observed phenotypes. This aligns with recent findings on Myc expression plasticity during clonal evolution in GBM.^51^ While Myc may be crucial for hybrid cell hypertranscription, our data show the nucleoplasmic transport machinery as the upstream effector, consistent with studies on NPC, chromatin, and protein import,^26,52,53^ suggesting NPC as the key regulator of the hybrid phenotype. Studies have explored targeting Myc’s oncogenic features in cancer cells^54^ with direct Myc inhibition under development.^55^ However, targeted therapy often fails against aggressive solid tumors due to cancer plasticity,^56^ thus targeting nucleoplasmic transport to reduce TF activity may offer an alternative approach.

The hybrid cell phenotype is epigenetically encoded, as we showed via the recently developed scChIP-Seq method.^21^ Notably, open chromatin is a key feature of stemness and reprogramming to pluripotent stem cells. Chromatin remodeling occurs during stem-like transitions and EMT in various cancers. A scRNA-Seq study on TGB-b-induced EMT in breast cancer revealed a intermediated EMT cell state remarkably similar to the hybrid signature,^57^ thus connecting our hybrid cells to a partial-EMT state.

Literature has long hypothesized that the low survival rate of GBM patients is due to GBM cell plasticity.^58^ Here, we investigated the molecular reasons for this, linking hybrid cell plasticity to open chromatin, nuclear pores, proliferation, and DDR machinery, causing therapy resistance. Our results on nucleoplasmic transport reveal new vulnerabilities in GBM cell plasticity, potentially leading to innovative treatments. These findings hold promise for future research and therapeutic development.

## Supplementary material

Supplementary material is available online at *Neuro-Oncology* (https://academic.oup.com/neuro-oncology).

noaf160_Supplementary_Materials

## Data Availability

RNA-seq, scRNA-seq and scChIP-seq data were deposited in NCBI’s Gene Expression Omnibus and are accessible through GSE256067. The mass spectrometry proteomics data have been deposited in the ProteomeXchange Consortium (http://proteomecentral.proteomexchange.org) via the PRIDE partner repository^59^ with the dataset identifier PXD050217. All other data are available from the corresponding author upon reasonable request.
