# Supplementary material for: Proneural–mesenchymal hybrid glioblastoma cells are resistant to therapy and dependent on nuclear import
Source: Neuro Oncol. 2025 Jul 8;27(11):2876–93. doi: 10.1093/neuonc/noaf160 (PMC12908486; doi:10.1093/neuonc/noaf160)
Supplement: noaf160_Supplementary_Materials [file noaf160_supplementary_materials.zip › noaf160_suppl_Supplementary-Figures.pdf]

Supplementary Information

Proneural-Mesenchymal hybrid glioblastoma cells are resistant to therapy and dependent on nuclear import

Authors

Guillaume Bourmeau[1], Oceane Anezo[1], Jeremy Raymond[2], Alberto Ballestín[1], Cathy Pichol-Thievend[1], Juliette Reveilles[1], Adrien Thomas[1], Lin Wang[3], Melanie Miranda[4,5,6], Eve Moutaux[4,5,6], Stephane Liva[7,8], Valentino Ribecco[1], Laetitia Besse[9], Florent Dingli[10], Damarys Loew[10], Celine Vallot[4,5,6], Gaetano Gargiulo[11], Vidhya M. Ravi[12], Kevin Joseph[12], Giorgio Seano[1,#]

Suppl Fig. 1

A.

MGG4	−4, −8p, −8q11.21-q24.13, ++8q24.21 **(MYC)**, −8q24.22-q24.23, ++8q24.24, −8q24.24, −10, −13q12.11-q21.31, +13q21.32-q33.4	Proneural
MGG8	−1p36.33-p34.3, +1q42.12-q44, ++2p24.3 (**MYCN**), +2p24.2-p13.1, +2q, +3, −4, ++4q12 (**PDGFRA**), +5q11.2-q31.1, −5q31.2-q35.3, +7, −8, −9p24.1-p21.1, −−9p21.3 (**CDKN2A&B**), +10p14-p11.22, −10p11.21, −10q, ++12q15 (**MDM2**), +12q21.1-q24.22, −13q12.11-q12.12, −13q13.3-q34, −−13q21.1, −14q12-q23.1, −15, +17p11.2, +17q, −18p, 18q11.2-q21.32, −−18q21.33-q23, +19p13.3-p13.1, −20, −21	Mesenchymal
P3	+7, +19, +20q, -1q42-43, -9, -10, -20p, --5q13.1 (**PIK3R1**), --9p21.3 (**CDKN2A&B**)	Classical

Abbreviations: + gain, − loss, ++ amplification, −− homozygous deletion.

B.

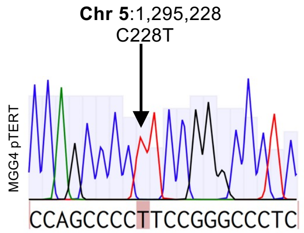

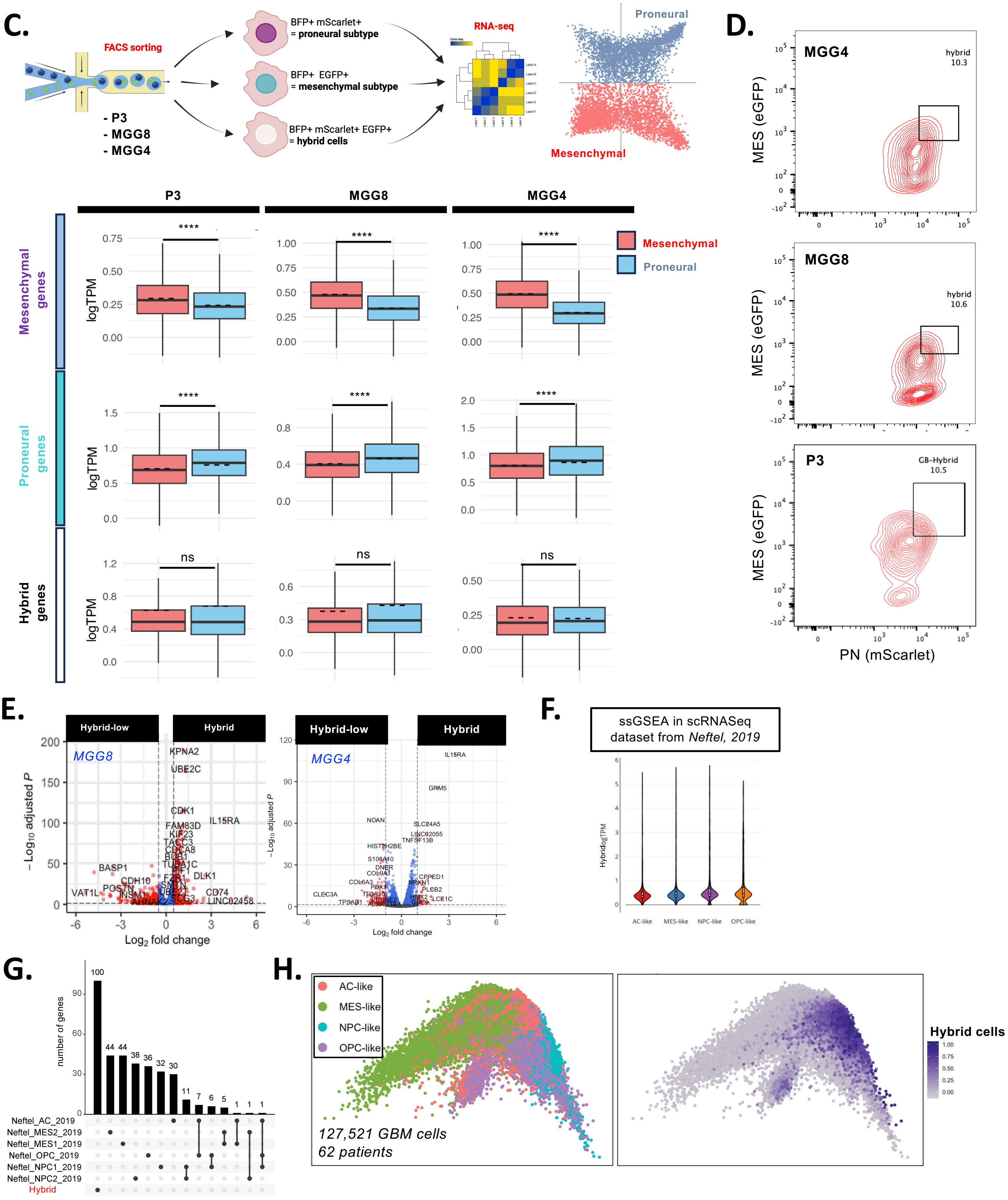

Figure S1

(A) Table of the mutational landscape of the cell lines used, along with their clinical subtype. **(B)** Sanger sequencing of MGG4 promoter showing the C228T mutation in this cell line. **(C)** ssGSEA of the top upregulated genes in sorted mesenchymal, proneural and hybrid populations using our reporters. **(Top)** Schematic of the experiment. For each cell line (MGG4, MGG8 and P3), proneural, mesenchymal and hybrid populations were sorted by cytometry, based on reporter intensity and RNA was sequenced. The expression of the differentially expressed genes (DEGs) of each subpopulation was mapped onto Neftel et al. cell states classification, divided in proneural and mesenchymal populations. **(Bottom)** The box plots represent the expression per cell of the DEGs of each cell line's sorted subpopulation (MES+, PN+ or hybrid) on Neftel et al. single cell dataset, where cells are divided in proneural or mesenchymal populations as depicted in the top right schematic. **(D)** Representative cytometry density plot of the reporter's fluorescence intensities in MGG4, P3 and MGG8 cell lines, made using FlowJo software. Hybrid cells are the top 10% cells expressing the most both MES and PN reporters. **(E)** Volcano plots of the differential expression analysis between Hybrid and Hybrid-low cells in MGG4 and MGG8. Vertical dotted bar is set at a fold change of 2, and horizontal bar represents a significance of p=0.05. **(F)** Hybrid signature score in TPM per cell state in Neftel et al. classification. **(G)** Upset plot showing the absence of overlap between Hybrid signature and Neftel et al. cell states signatures. **(H)** Feature plot for the Neftel cell states (left) and Hybrid signature (right) in the Proneural-Mesenchymal axis mapped using the Harmonized database (62 patients with GBM and 127.521 GBM cells in total)**.**

Suppl Fig. 2

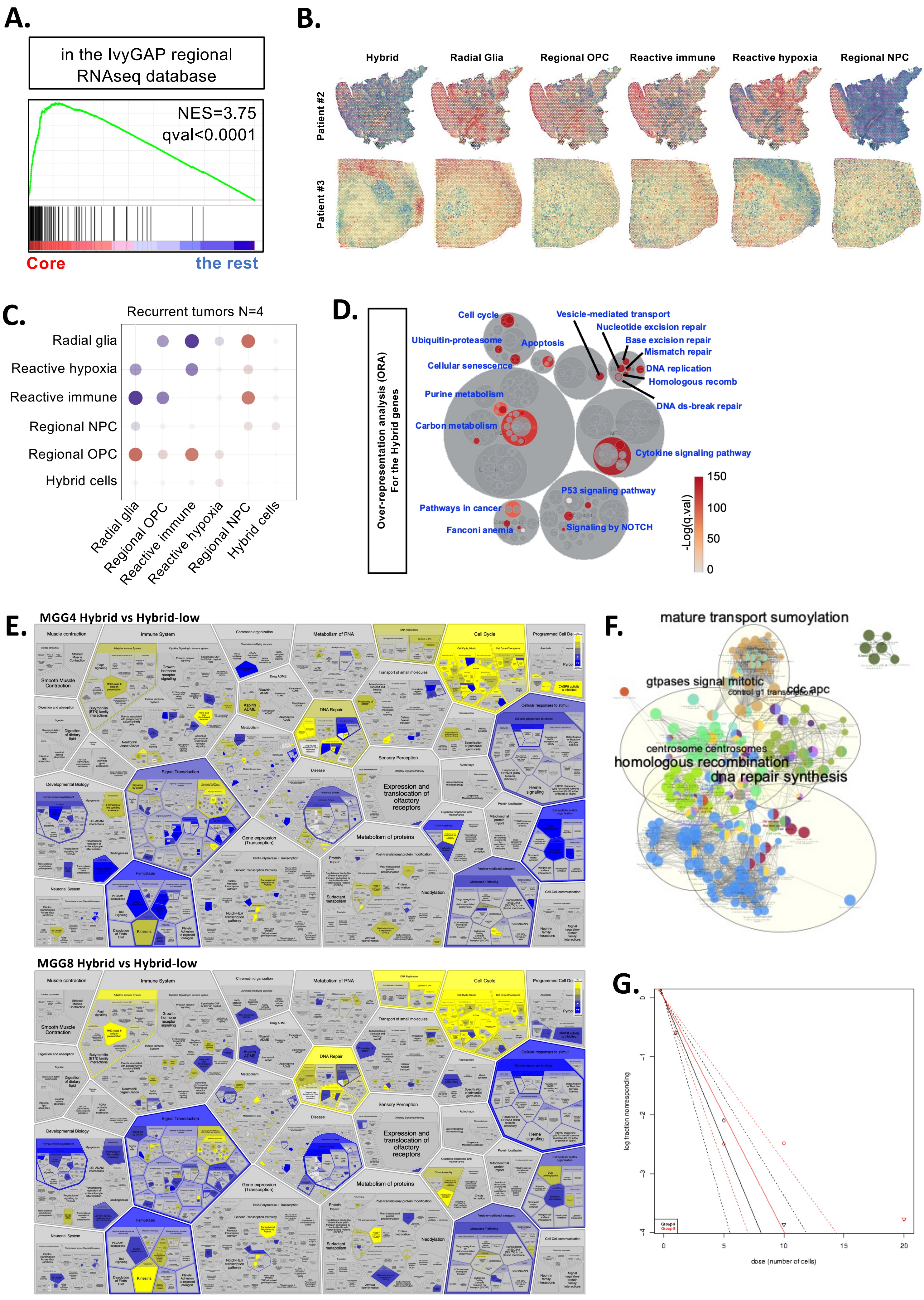
A.
in the IvyGAP regional RNAseq database
NES=3.75
qval<0.0001
Core
the rest
B.
Hybrid
Radial Glia
Regional OPC
Reactive immune
Reactive hypoxia
Regional NPC
Patient #2
Patient #3
C.
Recurrent tumors N=4
Radial glia
Reactive hypoxia
Reactive immune
Regional NPC
Regional OPC
Hybrid cells
D.
Over-representation analysis (ORA)
For the Hybrid genes
Cell cycle
Ubiquitin-proteasome
Apoptosis
Cellular senescence
Purine metabolism
Carbon metabolism
Vesicle-mediated transport
Nucleotide excision repair
Base excision repair
Mismatch repair
DNA replication
Homologous recomb
DNA ds-break repair
Cytokine signaling pathway
P53 signaling pathway
Pathways in cancer
Fanconi anemia
Signaling by NOTCH
-Log(q.val)
150
100
50
0
E.
MGG4 Hybrid vs Hybrid-low
MGG8 Hybrid vs Hybrid-low
F.
mature transport sumoylation
gtpases signal mitotic
control g1 transcription
cdc apc
centrosome centrosomes
homologous recombination
dna repair synthesis
G.
log fraction nonresponding
dose (number of cells)
0
-1
-2
-3
-4
0
5
10
15
20

Figure S2

(A) GSEA analysis of the IvyGAP atlas for the Hybrid cells signature. The core tumor is compared to the rest of the tumor regions. 122 RNA seq samples divided in 5 tumor regions from 10 patients. Normalized enrichment score (NES) and FDR q-value are indicated. **(B)** Representative surface plot of spatially resolved expression of Hybrid signature and spatial subtypes signatures in patients. Normalized GSEA score is color-coded with red dots being a high-expression of the corresponding signature**.** **(C)** Correlation plot between Hybrid signature and published GBM spatial subtypes in recurrent GBM patients. **(D)** Over-representation analysis for the upregulated genes in Hybrid cells. Significant pathways are colored and specified (performed using DecoPath). **(E)** Reactome over-representation analysis of MGG4 and MGG8 Hybrid vs Hybrid-low cells. The whole RNA-Seq result was used to perform the PADOG analysis (i.e., a weighted geneset analysis method that down-weighs genes that are present in many pathways). **(F)** Cytoscape annotation of Hybrid enriched pathways. Pathways enrichment was performed using the ClueGO pluging, and annotation of clusters of pathways was performed using the AutoAnnotate pluggin. **(G)** ELDA (Extreme Limiting Dilution Assay) assays of FACS-sorted Hybrid and Hybrid-low cells. The number of spheres was measured at days 7 and 14 and graph was generated using https://bioinf.wehi.edu.au/software/elda/. Plot is the log-fraction of the limiting dilution model. The slope of the line is the log-active fraction. The dotted lines give the 95% confidence interval.

Suppl Fig. 3

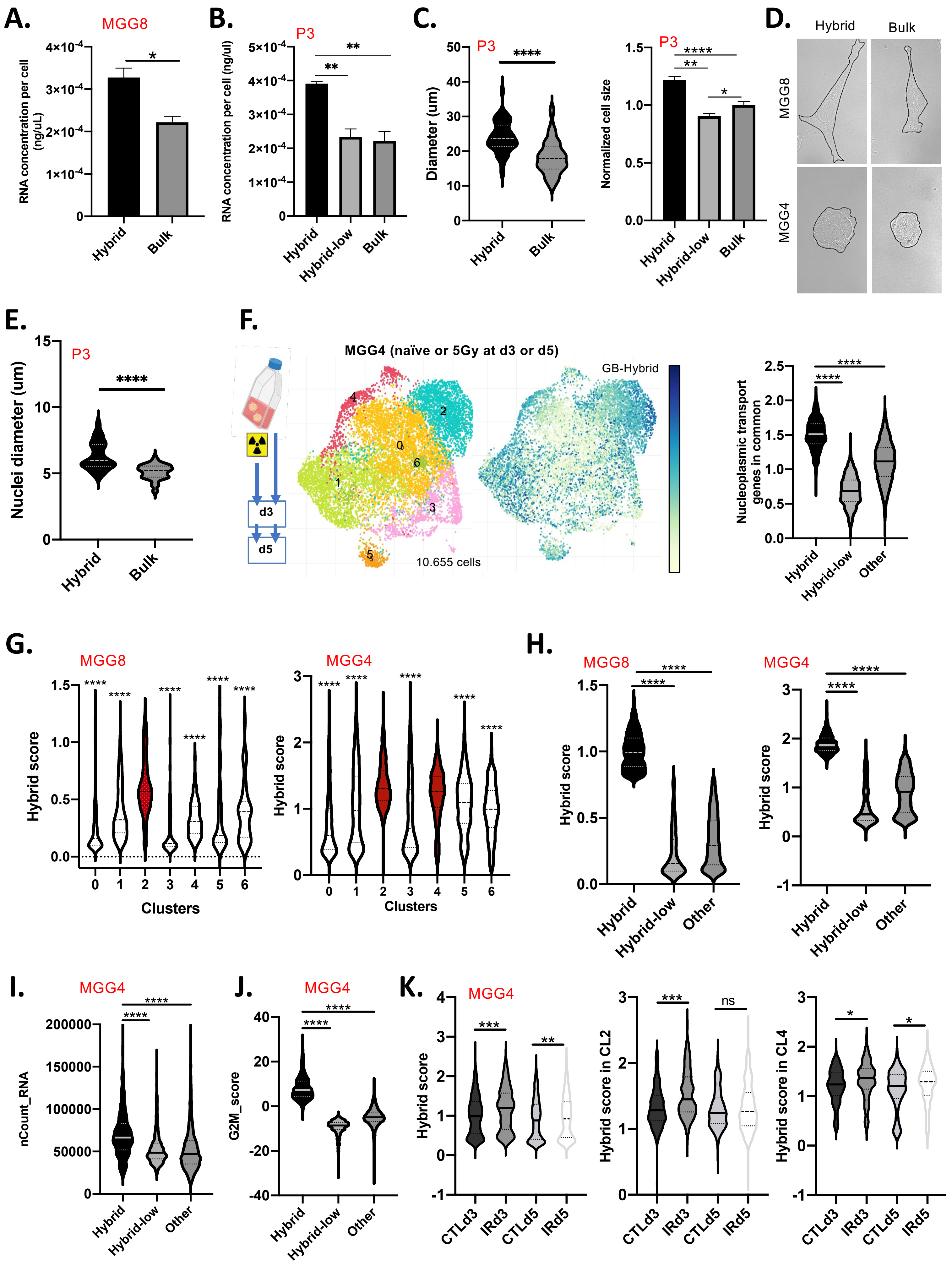
A.
MGG8
RNA concentration per cell (ng/uL)
Hybrid
Bulk
B.
P3
RNA concentration per cell (ng/ul)
Hybrid
Hybrid-low
Bulk
C.
P3
Diameter (um)
Normalized cell size
D.
Hybrid
Bulk
MGG8
MGG4
E.
P3
Nuclei diameter (um)
F.
MGG4 (naïve or 5Gy at d3 or d5)
d3
d5
GB-Hybrid
10.655 cells
Nucleoplasmic transport genes in common
Other
G.
MGG8
MGG4
Hybrid score
Clusters
H.
I.
nCount_RNA
J.
G2M_score
K.
Hybrid score in CL2
Hybrid score in CL4
CTLd3
IRd3
CTLd5
IRd5

Figure S3

(A) RNA quantity per cell in MGG8 Hybrid compared to bulk cells. (n=3; *p<0.05, paired t-tests) **(B)** RNA quantity per cell in P3 Hybrid compared to bulk and Hybrid-low cells. (n=3; **p<0.01, one-way ANOVA) **(C) (Left)** Diameter of P3 Hybrid and bulk cells on adherent cells. (n=150 cells examined over 3 independent experiments per condition. ****p<0.0001; unpaired t test). **(Right)** Normalized cell size of Hybrid, bulk and Hybrid-low P3 cells in 3D, measured by cytometry, using FSC measurements. (n=3; *p<0.05; **p<0.01; ****p<0.0001; one-way ANOVA). **(D)** Representative images of Hybrid size compared to bulk cells in 2D. **(E)** Size of nuclei in P3 Hybrid and bulk cells, measured on adherent cells. (n=1520 cells examined over 3 independent experiments per condition ****p<0.0001; unpaired t test). **(F) (Left)** MGG4 single-cell RNAseq. Cells were irradiated (5Gy) or not and sequenced 3 and 5 days later. UMAP dimensionality reduction resulted in 7 clusters. **(Middle)** Hybrid geneset expression in MGG4 scRNAseq. **(Right)** Hybrid cells are enriched in nuclear shuttling genes (Common nucleopore genes score) ****p<0.0001; one-way ANOVA. **(G)** Hybrid geneset score for each cluster in MGG8 scRNAseq (left) and MGG4 scRNAseq (right). The clusters in red have a significantly higher expression of hybrid geneset compared to the others. (****p<0.0001; one-way ANOVA). **(H)** Average Hybrid score after classification of cells in the different populations for MGG8 scRNAseq (left) and MGG4 scRNAseq (right) (****p<0.0001; one-way ANOVA). **(I)** RNA quantity per cell (n_Count_RNA) of hybrid Hybrid, Hybrid-low and the rest of the cells in MGG4 scRNAseq. ****p<0.0001; one-way ANOVA. **(J)** Proliferation index (G2M_score) of hybrid Hybrid, Hybrid-low and the rest of the cells in MGG4 scRNAseq. ****p<0.0001; one-way ANOVA. **(K)** Average hybrid geneset score for each experimental condition in MGG4 scRNAseq, for all the cells (left), cluster 2 (middle) and cluster 4 (right); ****p<0.0001; one-way ANOVA.

Suppl Fig. 4

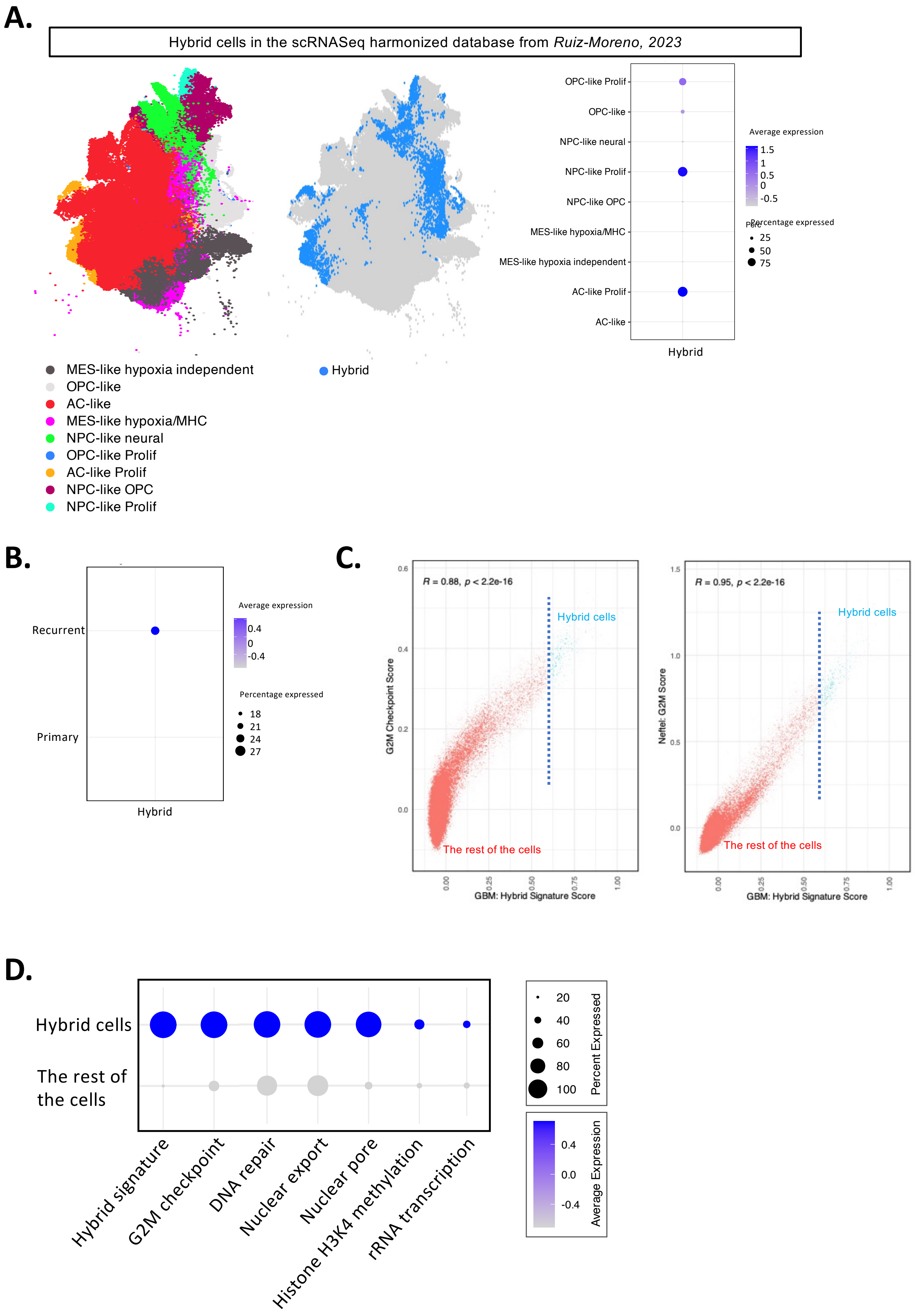

A.
Hybrid cells in the scRNASeq harmonized database from *Ruiz-Moreno, 2023*
OPC-like Prolif
OPC-like
NPC-like neural
NPC-like Prolif
NPC-like OPC
MES-like hypoxia/MHC
MES-like hypoxia independent
AC-like Prolif
AC-like
Hybrid
Average expression
1.5
1
0.5
0
-0.5
Percentage expressed
25
50
75
MES-like hypoxia independent
OPC-like
AC-like
MES-like hypoxia/MHC
NPC-like neural
OPC-like Prolif
AC-like Prolif
NPC-like OPC
NPC-like Prolif
Hybrid
B.
Recurrent
Primary
Hybrid
Average expression
0.4
0
-0.4
Percentage expressed
18
21
24
27
C.
R = 0.88, p < 2.2e-16
Hybrid cells
G2M Checkpoint Score
The rest of the cells
GBM: Hybrid Signature Score
R = 0.95, p < 2.2e-16
Hybrid cells
Neftel: G2M Score
The rest of the cells
GBM: Hybrid Signature Score
D.
Hybrid cells
The rest of the cells
Hybrid signature
G2M checkpoint
DNA repair
Nuclear export
Nuclear pore
Histone H3K4 methylation
rRNA transcription
20
40
60
80
100
Percent Expressed
0.4
0.0
-0.4
Average Expression

Figure S4

(A) Hybrid cells in the Harmonized database. **(Left)** Classification of neoplastic cells in according to the Neftel et al. cell states. **(Middle)** In blue, cells expressing highly Hybrid geneset. **(Right)** Dotplot of the expression of Hybrid geneset per cell state. **(B)** Dotplot of the expression of Hybrid geneset in primary of recurrent patient cells. Size indicates the proportion of cells expressing Hybrid geneset. **(C)** Correlation plots between Hybrid signature and Hallmark G2M checkpoint signature (left) or G2M signature (right). Each dot is an individual cell and the R correlation coefficient is indicated. Hybrid cells are defined as the top 10% cells with highest expression of Hybrid geneset. **(D)** Dotplot of the expression of Hallmark DNA repair pathway, nuclear shuttling pathways, G2M checkpoint, rRNA transcription and H3K4 methylation, in Hybrid versus the rest of the cells, showing an enrichment in all these pathways in Hybrid cells.

Suppl Fig. 5

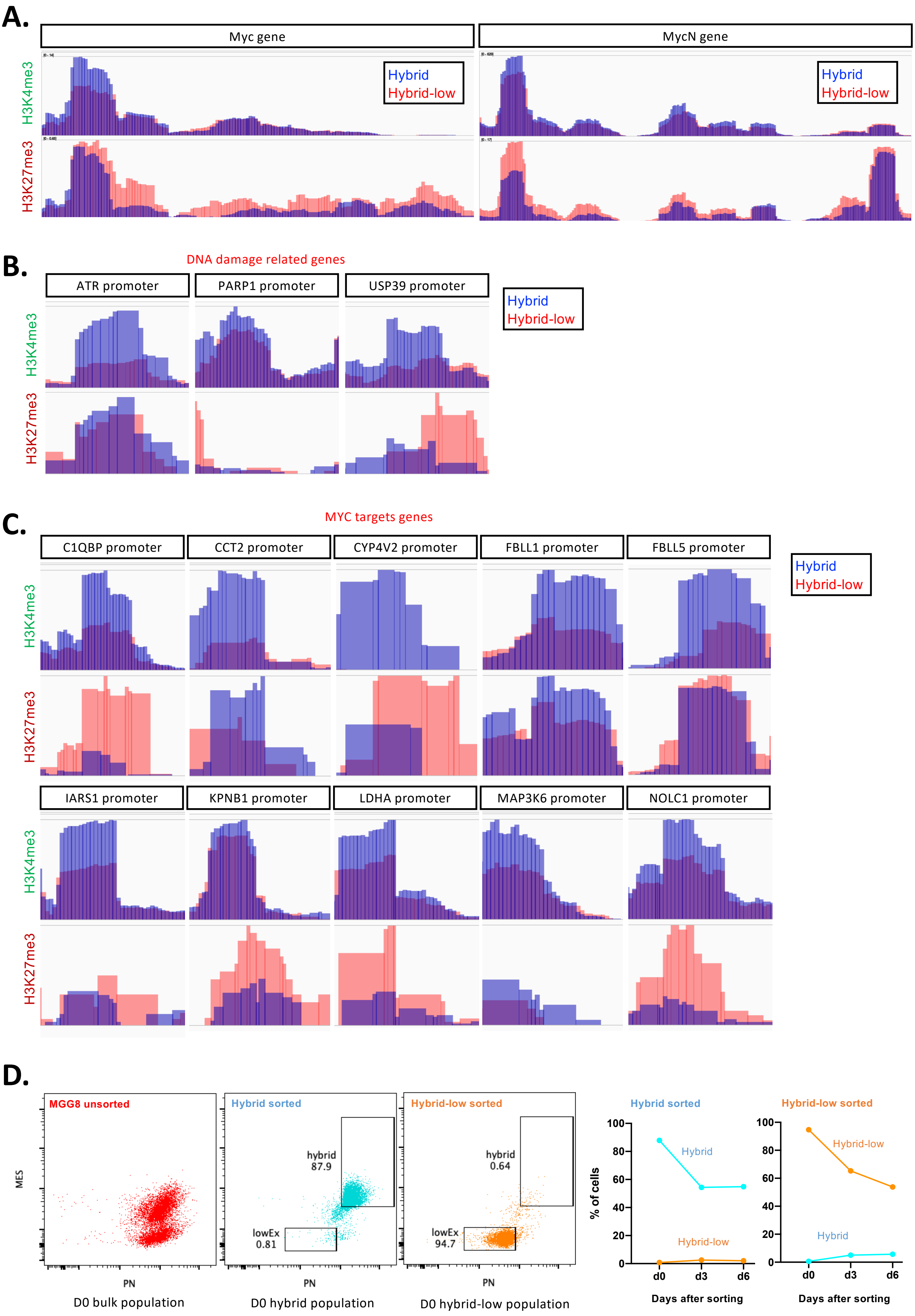

A.
Myc gene
MycN gene
Hybrid
Hybrid-low
H3K4me3
H3K27me3
B.
DNA damage related genes
ATR promoter
PARP1 promoter
USP39 promoter
Hybrid
Hybrid-low
H3K4me3
H3K27me3
C.
MYC targets genes
C1QBP promoter
CCT2 promoter
CYP4V2 promoter
FBLL1 promoter
FBLL5 promoter
Hybrid
Hybrid-low
H3K4me3
H3K27me3
IARS1 promoter
KPNB1 promoter
LDHA promoter
MAP3K6 promoter
NOLC1 promoter
H3K4me3
H3K27me3
D.
MGG8 unsorted
Hybrid sorted
Hybrid-low sorted
MES
hybrid
87.9
lowEx
0.81
hybrid
0.64
lowEx
94.7
PN
PN
PN
D0 bulk population
D0 hybrid population
D0 hybrid-low population
Hybrid sorted
Hybrid-low sorted
% of cells
100
80
60
40
20
0
Hybrid
Hybrid-low
d0
d3
d6
Days after sorting
100
80
60
40
20
0
Hybrid-low
Hybrid
d0
d3
d6
Days after sorting

Figure S5

(A) ChIPseq profiles of Hybrid (blue) and Hybrid-low (red) at MYC and MYCN gene loci, for H3K4me3 and H3K27me3 histone marks. **(B)** ChIPseq profiles of Hybrid (blue) and Hybrid-low (red) at the indicated loci of DNA damage related genes, for H3K4me3 and H3K27me3 histone marks. **(C)** ChIPseq profiles of Hybrid (blue) and Hybrid-low (red) at the indicated loci of MYC target genes, for H3K4me3 and H3K27me3 histone marks. **(D)** Plasticity of Hybrid and Hybrid-low cells with time. Populations were isolated and stained, mixed back in bulk population and analyzed over time using FACS. In red bulk, blue Hybrid and in orange Hybrid-low cells.

Suppl Fig. 6

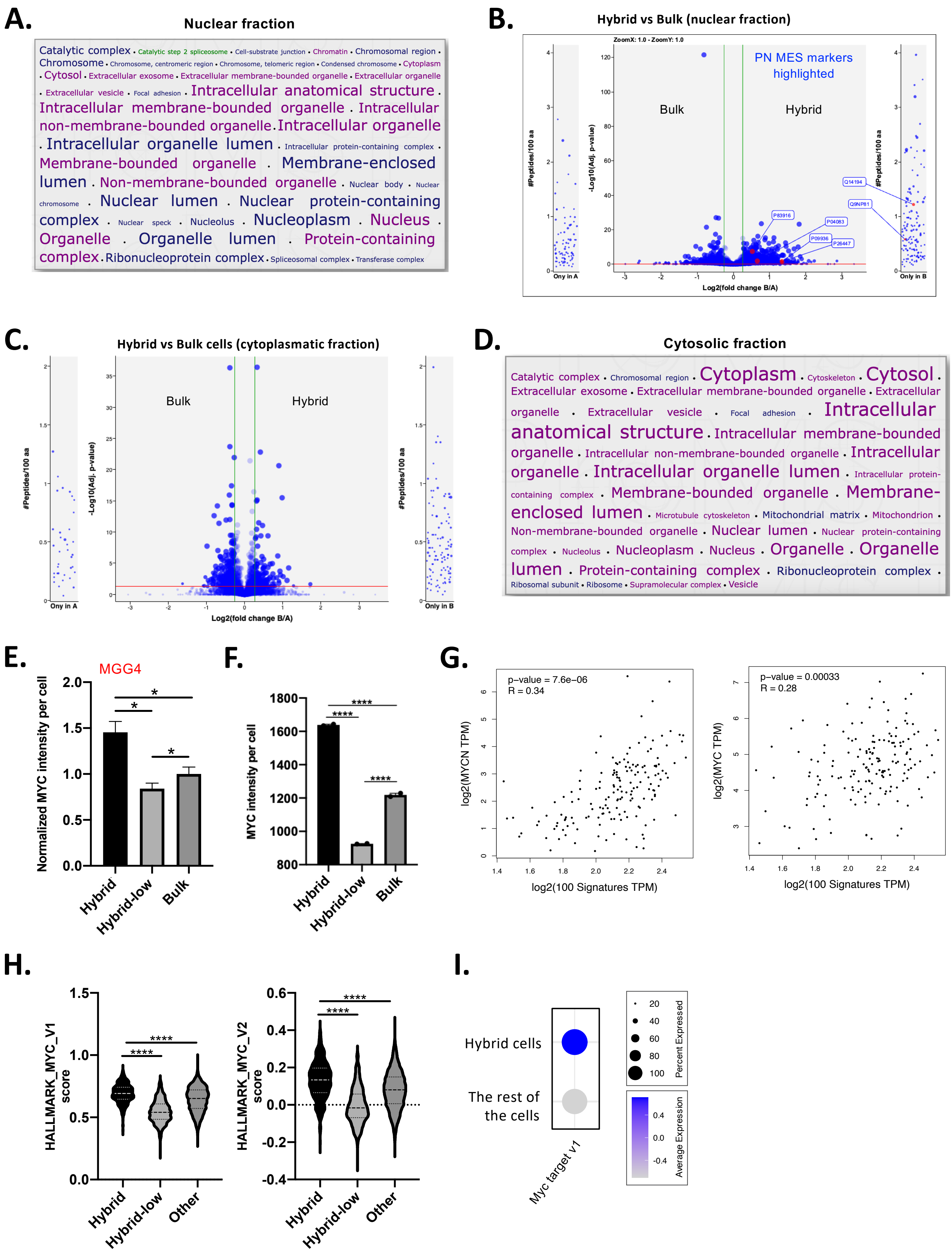
A.
Nuclear fraction
Catalytic complex • Catalytic step 2 spliceosome • Cell-substrate junction • Chromatin • Chromosomal region • Chromosome • Chromosome, centromeric region • Chromosome, telomeric region • Condensed chromosome • Cytoplasm • Cytosol • Extracellular exosome • Extracellular membrane-bounded organelle • Extracellular organelle • Extracellular vesicle • Focal adhesion • Intracellular anatomical structure • Intracellular membrane-bounded organelle • Intracellular non-membrane-bounded organelle • Intracellular organelle • Intracellular organelle lumen • Intracellular protein-containing complex • Membrane-bounded organelle • Membrane-enclosed lumen • Non-membrane-bounded organelle • Nuclear body • Nuclear chromosome • Nuclear lumen • Nuclear protein-containing complex • Nuclear speck • Nucleolus • Nucleoplasm • Nucleus • Organelle • Organelle lumen • Protein-containing complex • Ribonucleoprotein complex • Spliceosomal complex • Transferase complex
B.
Hybrid vs Bulk (nuclear fraction)
ZoomX: 1.0 - ZoomY: 1.0
PN MES markers highlighted
Bulk
Hybrid
#Peptides/100 aa
-Log10(Adj. p-value)
Log2(fold change B/A)
Ony in A
Only in B
Q14194
Q9NP81
P83916
P04083
P09936
P26447
C.
Hybrid vs Bulk cells (cytoplasmatic fraction)
Bulk
Hybrid
#Peptides/100 aa
-Log10(Adj. p-value)
Log2(fold change B/A)
Only in A
Only in B
D.
Cytosolic fraction
Catalytic complex • Chromosomal region • Cytoplasm • Cytoskeleton • Cytosol • Extracellular exosome • Extracellular membrane-bounded organelle • Extracellular organelle • Extracellular vesicle • Focal adhesion • Intracellular anatomical structure • Intracellular membrane-bounded organelle • Intracellular non-membrane-bounded organelle • Intracellular organelle • Intracellular organelle lumen • Intracellular protein-containing complex • Membrane-bounded organelle • Membrane-enclosed lumen • Microtubule cytoskeleton • Mitochondrial matrix • Mitochondrion • Non-membrane-bounded organelle • Nuclear lumen • Nuclear protein-containing complex • Nucleolus • Nucleoplasm • Nucleus • Organelle • Organelle lumen • Protein-containing complex • Ribonucleoprotein complex • Ribosomal subunit • Ribosome • Supramolecular complex • Vesicle
E.
MGG4
Normalized MYC intensity per cell
Hybrid
Hybrid-low
Bulk
F.
MYC intensity per cell
Hybrid
Hybrid-low
Bulk
G.
p–value = 7.6e–06
R = 0.34
log2(MYCN TPM)
log2(100 Signatures TPM)
p–value = 0.00033
R = 0.28
log2(MYC TPM)
log2(100 Signatures TPM)
H.
HALLMARK_MYC_V1 score
Hybrid
Hybrid-low
Other
HALLMARK_MYC_V2 score
Hybrid
Hybrid-low
Other
I.
Hybrid cells
The rest of the cells
Myc target v1
Percent Expressed
Average Expression

Figure S6

(A) GO term enrichment of nuclear fraction samples, showing an overrepresentation of nuclear-related terms. **(B)** Volcano plot for nuclear proteome analysis between Hybrid and bulk in MGG8 cells. Horizontal red line represents a significance level of p-value < 0.05. Vertical green bars are set to a fold change of 2. PN and MES markers mentioned are highlighted. **(C)** Volcano plots for cytoplasmic proteome analysis between Hybrid and bulk in MGG8 cells. Horizontal red line represents a significance level of p-value < 0.05. Vertical green bars are set to a fold change of 2. **(D)** GO term enrichment of cytoplasmic fraction samples, showing an overrepresentation of cytoplasm-related terms. **(E)** Normalized MYC intensity per cell in MGG4 Hybrid, bulk and Hybrid-low measured by cytometry (n=4; *p<0.05; **p<0.01; one-way ANOVA). **(F)** Normalized MYC intensity per cell in P3 Hybrid, bulk and Hybrid-low measured by cytometry (n=2; ****p<0.0001; one-way ANOVA). **(G)** Correlation analysis of Hybrid signature and MYC/MYCN in glioblastoma patients, showing a significant correlation. Performed in GEPIA2 (http://gepia2.cancer-pku.cn/#correlation). **(H)** Average hallmark genesets MYC_V1 and MYC_V2 scores per cell in MGG8 scRNAseq, per population. Hybrid have a higher score than the other populations. **(I)** Dotplot showing the higher expression of MYC_V1 geneset in patient GBM cells of the harmonized database.

Suppl Fig. 7

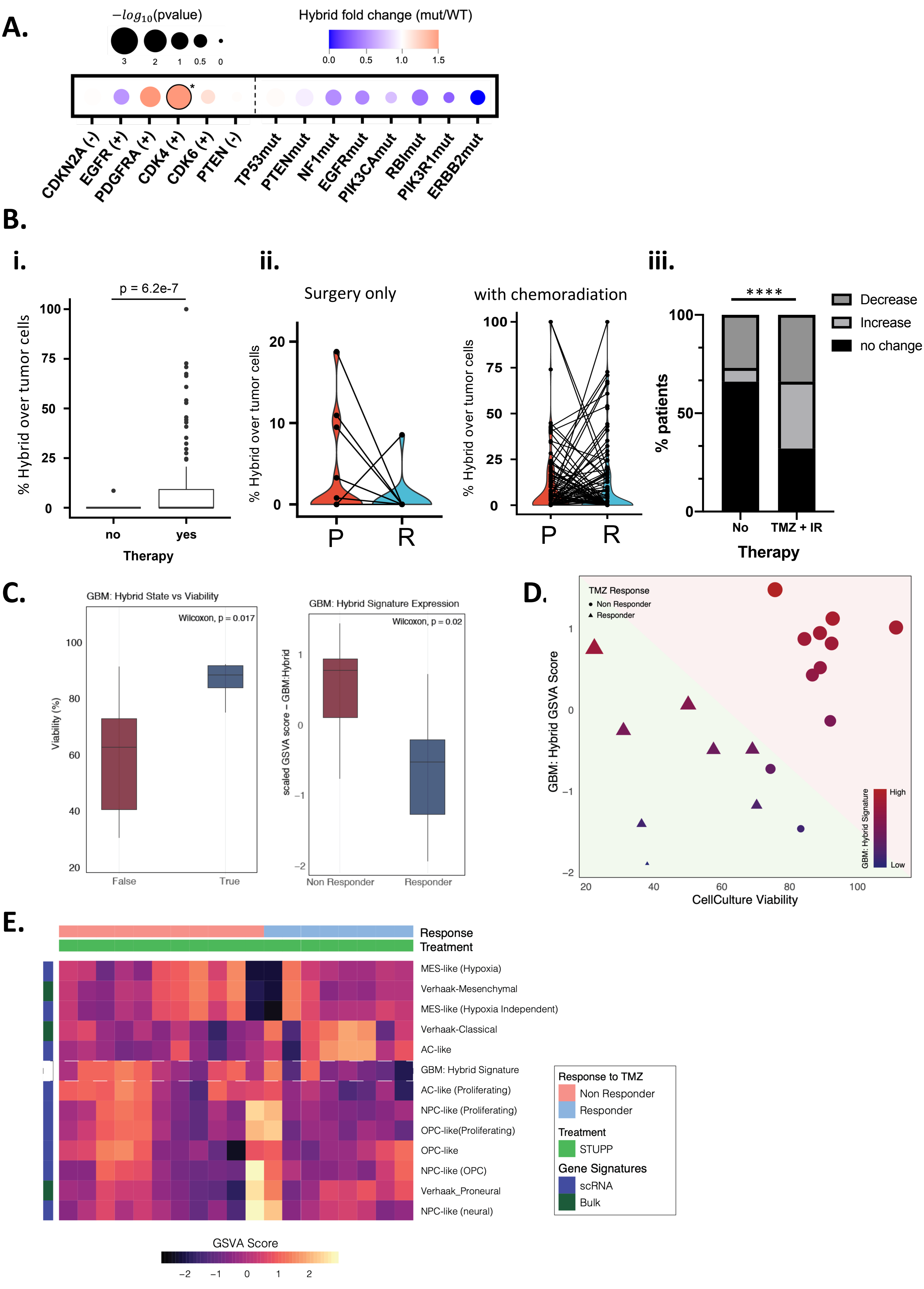

A.
$-log_{10}$(pvalue)
Hybrid fold change (mut/WT)
CDKN2A (-)
EGFR (+)
PDGFRA (+)
CDK4 (+)
CDK6 (+)
PTEN (-)
TP53mut
PTENmut
NF1mut
EGFRmut
PIK3CAmut
RBImut
PIK3R1mut
ERBB2mut
B.
i.
p = 6.2e-7
% Hybrid over tumor cells
Therapy
no
yes
ii.
Surgery only
with chemoradiation
% Hybrid over tumor cells
P
R
iii.
****
Decrease
Increase
no change
% patients
No
TMZ + IR
Therapy
C.
GBM: Hybrid State vs Viability
Wilcoxon, p = 0.017
Viability (%)
False
True
GBM: Hybrid Signature Expression
Wilcoxon, p = 0.02
scaled GSVA score – GBM:Hybrid
Non Responder
Responder
D.
TMZ Response
Non Responder
Responder
GBM: Hybrid GSVA Score
CellCulture Viability
GBM: Hybrid Signature
High
Low
E.
Response
Treatment
MES-like (Hypoxia)
Verhaak-Mesenchymal
MES-like (Hypoxia Independent)
Verhaak-Classical
AC-like
GBM: Hybrid Signature
AC-like (Proliferating)
NPC-like (Proliferating)
OPC-like(Proliferating)
OPC-like
NPC-like (OPC)
Verhaak_Proneural
NPC-like (neural)
Response to TMZ
Non Responder
Responder
Treatment
STUPP
Gene Signatures
scRNA
Bulk
GSVA Score

Figure S7

(A) Correlation between Hybrid cells and genetics aberrations. Using the GLASS dataset, the proportion of hybrid cells in primary tumors of patients with a given mutation or copy number variation was compared to the proportion of hybrid in primary tumors with wild type allele or normal ploidy. Size indicates log10 pvalue. Color indicates fold change. n=118; *p<0.05, Wilcoxon test. **(B)** Deconvolution analysis on patient data. **(i)** Percentage of Hybrid cells over tumor cells in recurrent tumors from the longitudinal GLASS dataset. Recurrent tumors are divided in two groups, patients that received chemoradiation before recurrence and patients that did not. There is a significant enrichment of Hybrid cells in the second group compared to the first. (p= 6.2e-7, t test). **(ii) (Left)** Evolution of Hybrid proportion in patients that received surgery only but no chemoradiation between primary (P) and recurrent (R) tumors. **(Right)** Evolution of Hybrid proportion in patients that received chemoradiation between primary (P) and recurrent (R) tumors. **(iii)** Percentage of patients with a reduction, an increase or no change in the proportion of Hybrid cells in their recurrent versus primary tumor. Patients are divided in two groups, patients that received chemoradiation before recurrence and patients that did not. Related to panel A.ii. ****p<0.0001, chi-square test. **(C)** Data mining for TMZ non-responders GBM cell lines (from Ntafoulis et al., 2023). **(Left)** Determination of the responders and non-responders cell lines. Responders (average viability after treatment =45%); non-responders (average viability after treatment =90%). **(Right)** Scaled Gene Set Variation Analysis (GSVA) for Hybrid signature after TMZ treatment in responders and non-responders. **(D)** Scatter plot of Hybrid signature enrichment vs Cell line viability. The region shaded in green represents samples that exhibited response to TMZ, vs the region in red which represents samples that exhibited low response to TMZ. There is an enrichment of the Hybrid Signature in the samples that exhibit resistance to TMZ. **(E)** Expression of Hybrid signature along with other published cell states from bulk and scRNA studies in the cell lines from Ntafoulis et al., 2023.

Suppl Fig. 8

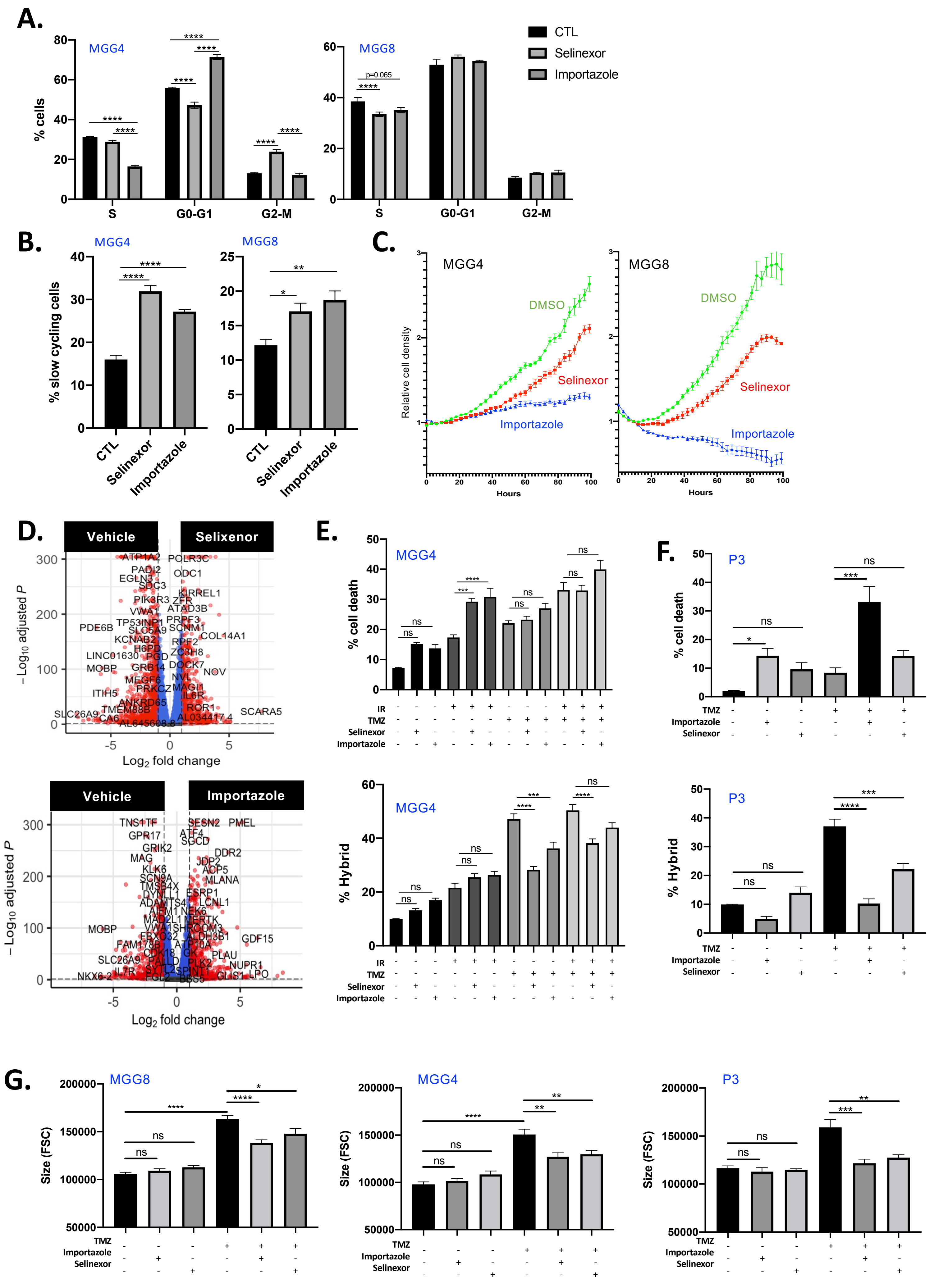
A.
MGG4
MGG8
% cells
S
G0-G1
G2-M
p=0.065
CTL
Selinexor
Importazole
B.
% slow cycling cells
C.
Relative cell density
DMSO
Hours
D.
Vehicle
Selixenor
Importazole
Log2 fold change
−Log10 adjusted P
E.
% cell death
% Hybrid
IR
TMZ
F.
P3
G.
Size (FSC)

Figure S8

(A) Proliferation analysis of MGG4 and MGG8 cells treated with Selinexor (100nM) and importazole (9uM) for three days using EdU incorporation and a DNA stain. The percentage of cells in S phase is reduced with drugs (n=4; ns, non-significant; **p<0.01; ***p<0.001; ****p<0.0001; 2way ANOVA). **(B)** Slow cycling cells proportion, defined by EdU positive signal 3 days after a 1-hour pulse of EdU, with or without nuclear shuttling drugs (n=4; ns, non-significant; **p<0.01; ***p<0.001; ****p<0.0001; one-way ANOVA). **(C)** Representative growth curves of MGG4 and MGG8 cells over time under nuclear shuttling inhibitors, using incucyte live-cell imaging system. **(D)** Volcano plots of the differential expression analyses between control cells and treated with Selinexor or Importazole. Vertical dotted bar is set at a fold change of 2, and horizontal bar represents a significance of p=0.05. **(E)** Combination of nuclear shuttling inhibitors and conventional therapies in MGG4. **(Top)** Cell death **(Bottom)** Hybrid percentage. IR 5Gy; TMZ 25uM; Selinexor 50nM; Importazole 9 uM (n=5; ns, non-significant; ***p<0.001; ****p<0.0001; one-way ANOVA). **(F)** Combination of nuclear shuttling inhibitors and temozolomide in P3. **(Top)** Cell death **(Bottom)** Hybrid percentage. TMZ 200uM; Selinexor 5uM; Importazole 20uM (n=3; ns, non-significant; *p<0.05; ***p<0.001; ****p<0.0001; one-way ANOVA). **(G)** 3D Cell size of MGG8 (left); MGG4 (middle), and P3 (right) cells, with or without therapies using the FSC measurement in cytometry. (MGG8: TMZ 10uM; Selinexor 100nM; Importazole 9uM; n=5. MGG4: TMZ 10uM; Selinexor 50nM; Importazole 9uM; n=4. P3: TMZ 200uM; Selinexor 5uM; Importazole 20uM; n=3). ns, non-significant; *p<0.05; **p<0.01; ***p<0.001; one-way ANOVA).

Suppl Fig. 9

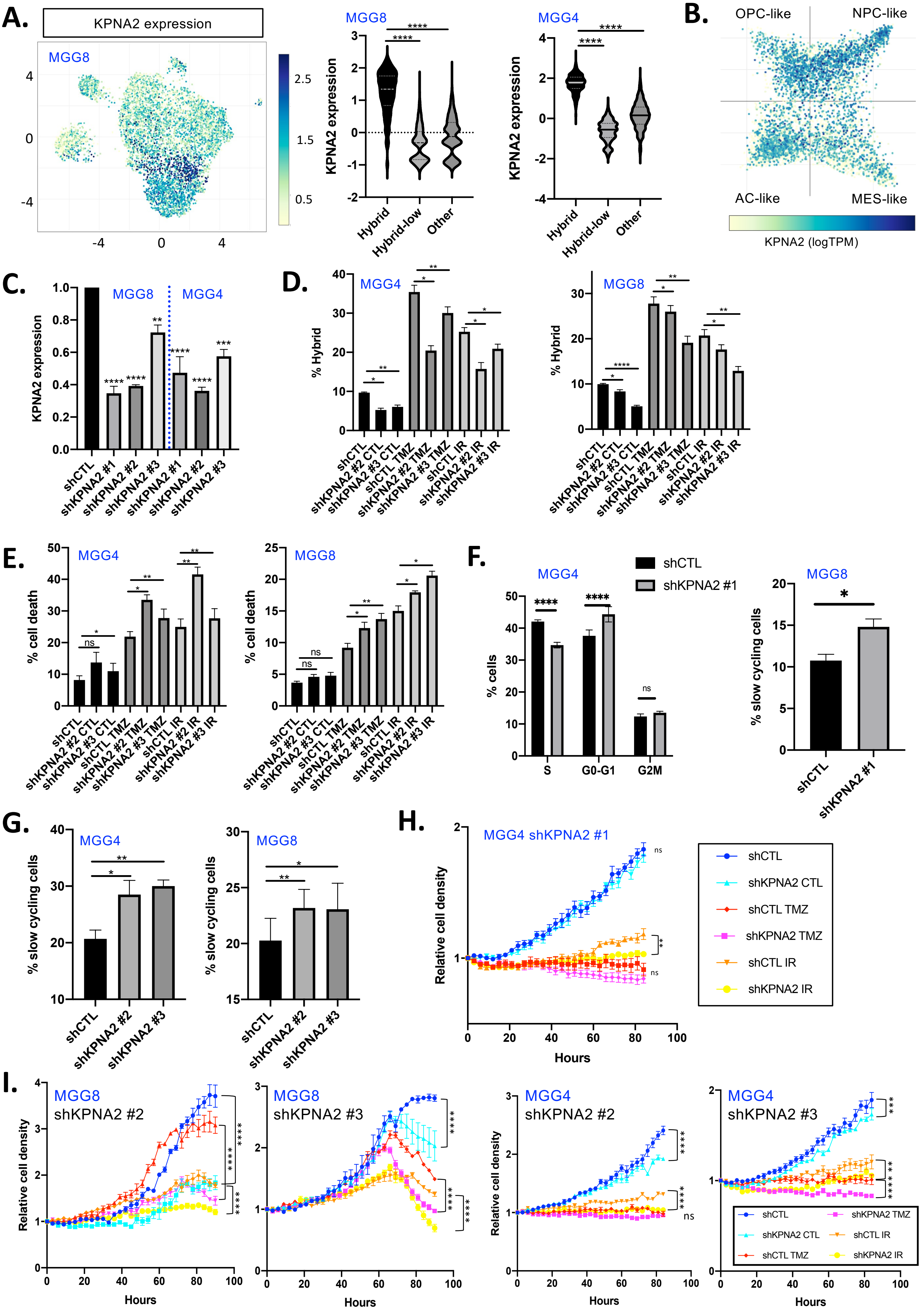

A.
KPNA2 expression
MGG8
MGG4
KPNA2 expression
Hybrid
Hybrid-low
Other
B.
OPC-like
NPC-like
AC-like
MES-like
KPNA2 (logTPM)
C.
KPNA2 expression
shCTL
shKPNA2 #1
shKPNA2 #2
shKPNA2 #3
D.
% Hybrid
shCTL
shKPNA2 #2 CTL
shKPNA2 #3 CTL
shCTL TMZ
shKPNA2 #2 TMZ
shKPNA2 #3 TMZ
shCTL IR
shKPNA2 #2 IR
shKPNA2 #3 IR
E.
% cell death
F.
shCTL
shKPNA2 #1
% cells
S
G0-G1
G2M
% slow cycling cells
G.
% slow cycling cells
H.
MGG4 shKPNA2 #1
Relative cell density
Hours
shCTL
shKPNA2 CTL
shCTL TMZ
shKPNA2 TMZ
shCTL IR
shKPNA2 IR
I.
MGG8 shKPNA2 #2
MGG8 shKPNA2 #3
MGG4 shKPNA2 #2
MGG4 shKPNA2 #3
Relative cell density
Hours

Figure S9

(A) KPNA2 expression in MGG8 and MGG4 scRNAseq. **(Left)** UMAP of KPNA2 expression in MGG8 scRNAseq, **(Middle)** KPNA2 expression in Hybrid, Hybrid-low or the rest of the cells (****p<0.0001; one-way ANOVA) in MGG8 scRNAseq. **(Right)** KPNA2 expression in Hybrid, Hybrid-low or the rest of the cells (****p<0.0001; one-way ANOVA) in MGG4 scRNAseq. **(B)** KPNA2 expression in Neftel et al. database. Cell states are indicated. **(C)** Efficiency of KPNA2 knock down in MGG4 and MGG8 cell lines by qPCR (n=3; **p<0.01; ***p<0.001; ****p<0.0001; one-way ANOVA). **(D)** Cytometry analysis of the Hybrid cells proportion in control, shKPNA2 #2 and shKPNA2 #3 in MGG4 (left) and MGG8 (right) cells basally and after treatments (5Gy IR or 25uM TMZ for MGG4, 5uM for MGG8). (n=4 in MGG4 n=5 in MGG8; ns, non-significant; *p<0.05; **p<0.01; ****p<0.0001; paired t-test). **(E)** Cell death in MGG4 (left) and MGG8 (right) cells is increased after therapies when KPNA2 is knock-down with shKPNA2 #2 and #3. (n=4 in MGG4 n=5 in MGG8; ns, non-significant; *p<0.05; **p<0.01; ****p<0.0001; paired t-test). **(F)** **(Left)** EdU assay of MGG4 showing a decreased proliferation with KPNA2 knock-down. (n=5; ns, non-significant; ****p<0.0001; 2way ANOVA) **(Right)** Slow cycling cells proportion, defined by EdU positive signal 2 days after a 1-hour pulse of EdU. MGG8 cells shows a slower proliferation in shKPNA2 #1 cells. (n=4; *p<0.05; paired t-test). **(G)** Slow cycling cells proportion, defined by EdU positive signal 2 days after a 1-hour pulse of EdU. Both MGG4 and MGG8 cells shows a slower proliferation in shKPNA2 #2 and #3 cells (n=4; *p<0.05; **p<0.01; paired t-test). **(H)** Representative growth curves of MGG4 short hairpin control and shKPNA2 #1 cells over time after conventional therapies (IR 5Gy, TMZ 25uM), using incucyte live-cell imaging system (n=3, ns, non-significant; two-way ANOVA on end-point is indicated). **(I)** Representative growth curves of short hairpin control, shKPNA2 #2 and shKPNA2 #3 cells over time after conventional therapies in MGG8 (IR 5Gy, TMZ 5uM) and MGG4 (IR 5Gy, TMZ 25uM), using incucyte live-cell imaging system (n=3, ns, non-significant; **p<0.01; ***p<0.001; ****p<0.0001; two-way ANOVA on end-point is indicated).

Suppl Fig. 10

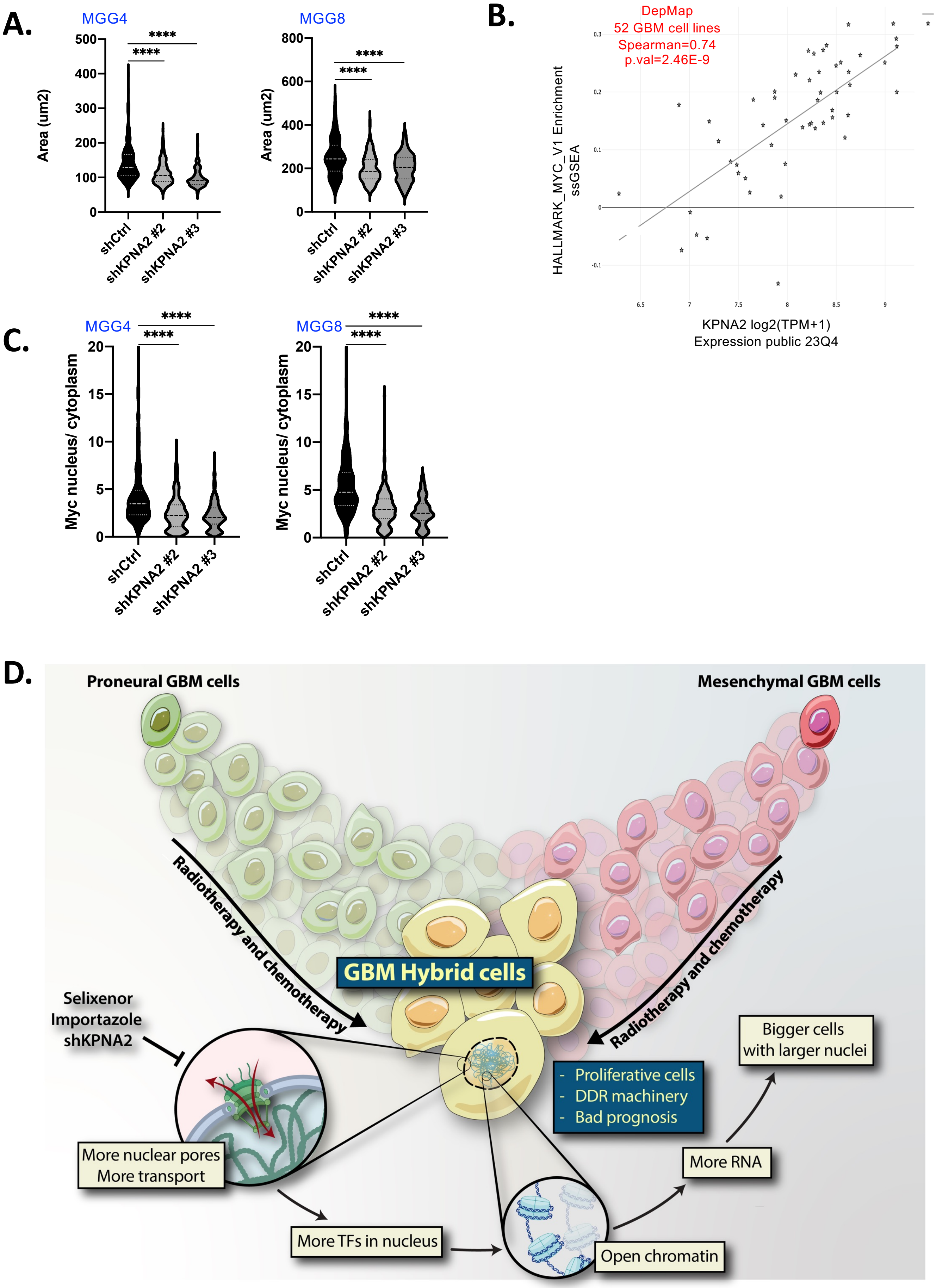

A.
MGG4
MGG8
Area (um2)
****
shCtrl
shKPNA2 #2
shKPNA2 #3
B.
DepMap
52 GBM cell lines
Spearman=0.74
p.val=2.46E-9
HALLMARK_MYC_V1 Enrichment
ssGSEA
KPNA2 log2(TPM+1)
Expression public 23Q4
C.
MGG4
MGG8
Myc nucleus/ cytoplasm
****
shCtrl
shKPNA2 #2
shKPNA2 #3
D.
Proneural GBM cells
Mesenchymal GBM cells
Radiotherapy and chemotherapy
GBM Hybrid cells
Radiotherapy and chemotherapy
Selixenor
Importazole
shKPNA2
Bigger cells
with larger nuclei
- Proliferative cells
- DDR machinery
- Bad prognosis
More nuclear pores
More transport
More RNA
More TFs in nucleus
Open chromatin

Figure S10

(A) Cell size of shKPNA2 #2 and shKPNA2 #3 cells compared to a control short hairpin in 2D by imaging, quantified as cell area, for MGG4 and MGG8 cell lines. (at least 150 cells per condition were examined over 3 independent experiments; ns, non-significant; ****p<0.0001; one-way ANOVA). **(B)** Correlation analysis between the expression of KPNA2 and the Hallmark MYC signature in 52 GBM cell lines. Performed using https://depmap.org/portal/. **(C)** MYC subcellular quantification in shKPNA2 #2 and shKPNA #3 cells by imaging, in both MGG4 and MGG8 cell lines. Quantifications are presented as ratios of MYC intensity between nuclei and cytoplasm. (at least 150 cells per condition were examined over 3 independent experiments; ****p<0.0001, one-way ANOVA). **(D)** Graphical abstract.

Supplementary material and methods

Cell lines

GBM patient-derived cell lines MGG4 and MGG8 from Dr. Wakimoto (Dept. of Neurosurgery, Massachusetts General Hospital, USA) and P3 from Dr. Daubon (IBGC, Bordeaux, France) wer cultured as neurospheres in NeuroCult basal medium supplemented with NeuroCult proliferation supplement (STEMCELL Technologies, #05751), EGF and bFGF at 20ng/ml, 5ug/ml heparin (STEMCELL Technologies, #07980), and gentamycin (Sigma), in low-attachment flasks (Thermo Scientific, #174952). Cells, negative for mycoplasma (Mycoplasma Detection Kit, MB Minerva Biolabs, #117048) were authenticated and mantained for a maximum of 15 passages undergoing passaging at 100-150 μm diameter or upon reaching high density, using Accutase (Thermo Fisher Scientific, A11105-01).

Plasmids, lentivirus production and inhibitors

Selinexor (No.S7252) and importazole (No.S8446) were purchased from Selleckem. The validated KPNA2 silencing vector were purchased from Sigma (TRCN0000293910), TCATGTAGCTGAGACATAAAT; (TRCN0000286475), GCTGGTTTGATTCCGAAATTT; (TRCN0000382469), TGTGGGCCGTGACCAACTATA. Lentiviral particles were generated by co-transfecting the above lentiviral vectors with plasmids pCMV-8.1 and VSVG into HEK293T cells. Transduced cells underwent selection with 0.25 μg/mL puromycin 48 hours post-transduction for at least 10 days.

RT-qPCR

Total RNA was extracted using the QIAGEN RNeasy Mini kit (#74104) according to the manufacturer's instructions, including a DNase digestion step. RNA concentration and quality were determined using a NanoDrop spectrophotometer (Thermo Fisher). cDNA was generated using 1ug of RNA and superscript III (#18080044; Thermo Fisher), followed by Taqman assay-based qPCR with QuantStudio5 (Applied Biosystems) using the Fast Advanced Master Mix (#4444556; Thermo Fisher).

In vitro irradiation and drug treatments

Neurospheres were collected by centrifugation and dissociated using Accutase. Cells were counted, seeded at required densities, and irradiated at the indicated dose using GSR Cs137/C

equipped with a cesium source (Gamma Service Medical GmbH). For TMZ treatment, cells were seeded and TMZ (diluted in DMSO) was added at the indicated concentrations.
For combinatory experiments with selinexor and importazole, the drugs were added at the indicated concentrations after seeding, at the same time of IR and TMZ.

FACS sorting

Neurospheres were centrifuged, dissociated into single cells using accutase, washed with PBS and centrifuged again. Pellet was resuspended in Neurocult medium and filtered in a FACS tube (Falcon, #352235). Cells were sorted using a FACSAria^{TM}III sorter (BD Biosciences) at 4°C using the following gating strategy. Viable cells were gated based on FSC-A/SSC-A, and doublets were excluded using the FSC-H/FSC-A and SSC-H/SSC-A parameters. The resulting single-cell healthy population was either sorted as a whole for bulk population or sorted depending on the FITC and PE-A parameters for hybrid and hybrid-low cells. Hybrid cells were the 10% cells with the highest fluorescence intensity of both parameters and hybrid-low the 10% cells with the lowest intensity of both parameters.

FACS analysis

Hybrid, cell death and size. For each experiment, on the day of FACS analysis, the cells were collected, dissociated with accutase and resuspended in Neurocult medium containing 1 uM of far red sytox (Thermo Fisher, S11380). The cells were incubated 15 min at 37°C, washed and resuspended in MACS buffer (Miltenyi Biotec) for FACS analysis. For all experiments, analyses were performed using FlowJo 10.8.2 software (BD Biosciences).
For hybrid cells proportion analysis, live cells were gated and doublets were excluded. Cells were displayed based on FITC and PE-A channels, and cells with the highest fluorescence in both channels were gated as hybrid cells. In the case of time-course analysis upon treatment, this gate was fixed to measure the proportion of hybrid cells in condition to the therapy conditions.
For cell size, live single cells were gated and mean FSC-A was scored.
For cell death, debris were excluded using the FSC-A/SCC-A channels, and dead cells were detected with Alexa-647 channel for far red sytox.
Cell cycle. Cells were seeded at 1x10^6 cells/well in 6-well plates. 24 hours later 10uM EdU was added for 1 hour at 37°C. Cells were then dissociated as described previously and sorted according to their reporter fluorescence intensity. The Click-iT EdU Alexa Fluor 647 kit (Thermo Fisher #C10419) was used according to the manufacturer’s instructions.

EdU incorporation in actively cycling cells was then detected, and 1uM blue sytox (ThermoFisher #S34857) was added to the tubes a few minutes before analysis for detection of DNA quantity.

Alternatively, to compare the cell cycle speed, cells were seeded and incubated with EdU for one hour. Cells were then washed and after two or three days, Click-iT EdU Alewa Fluor 647 kit was used.

Immunolabelling. Histone: cells were dissociated as described, washed, fixed using 4% PFA for 15 min, and permeabilized by the addition of 100% ice-cold methanol to cells in PBS to reach a 90% methanol final concentration. After 15 min cells were washed and cells were incubated for 1 h at room temperature with 1:1000 primary antibody for H3K4me3 (Cell Signaling Technologies, #9751). The cells were then washed and incubated for 30 min at room temperature with 1:500 Alexa Fluor 647 secondary antibody. Finally, the cells were resuspended in the MACS buffer for FACS analysis.

MYC: cells were dissociated, washed, fixed using 4% PFA for 15 min and permeabilized using PBS 0.3% Triton X-100 for 15 min. Cells were then blocked using PBS, 2% BSA, 0.1% Tween20 for 45 min. Primary antibody against Myc (Cell Signaling Technology, 13987) was incubated at 1:400 for one hour at room temperature in blocking buffer. Cells were washed and incubated with 1:500 Alexa Fluor 647 antibody for 45 min at room temperature. Finally, cells were washed and resuspended in PBS for cytometry processing.

Immunofluorescence of Myc and quantification

30,000 cells were seeded on Collagen-I matrix (50ug/ml) in an Ibidi chamber slide (m-slide 8well Ibidi GmbH, Germany, #80826) for 3h. Cells were then fixed with 4% PFA for 15 minutes, permeabilized and blocked using PBS completed with Triton X-100 0,1% and FBS 10% for 1h. Cells were incubated overnight with primary antibody against c-Myc/N-Myc (1:400, D3N8F Rabbit mAb, Ozyme, #13987S) in the blocking buffer at 4°C. Cells were then washed in PBS and incubated with Alexa Fluor 647-conjugated secondary antibody (1:1000; Thermo Fisher Scientific; #A-21244) for 1h at RT. Images were acquired using a widefield inverted microscope (Leica DMI 6000B) at 40X magnification (dry objective with plan Apo correction and NA: 0.95). Fluorescence was collected on an ORCA-Fusion Digital sCMOS camera with a far-red bloc filters (Excitation BP 645/30; Emission BP 705/72). Nuclear versus cytoplasmic Myc signal was analyzed using a custom ImageJ macro designed to quantify the signal in each compartment.

Size of cells

80,000 cells were seeded on a 12-well plate coated with 50ug/ml of Collagen-I for 1 h to reach 70% confluency. An inverted widefield Leica microscope with 10X magnification was used for

image acquisition at 37°C and 5% CO2. Multistage positions with 1.6um calibration (x/y) were acquired using a brightfield wavelength.

For size of cells, elliptical tools in ImageJ were used to measure the diameter of cells and freehand line tool was allowed to draw the area of cells.

Size of nuclei

Cells were dissociated and sorted into the required populations as described. $5x10^5$ cells were resuspended in 1250 ul Neurocult media at 10uM DRAQ5 concentration. Cells were incubated at 37°C for 3 min. Cells were washed, centrifuged, resuspended in 100uL of 1% LM agarose at 37°C, and added to u-slide wells (Ibidi, #81826). 3D live confocal images (z step size: 0.1 µm) were acquired with a Nipkow Spinning Disk confocal system (Yokogawa CSU-X1-A1) mounted on a Nikon Eclipse Ti E inverted microscope, equipped with a 60x Apochromat oil-immersion objective (NA: 1.49) and captured on sCMOS Prime 95B camera (Photometrics) operated through MetaMorph® software (Molecular Devices). 3D reconstruction and nuclei volume measurements were performed using surface module on Bitplane Imaris software (Oxford Instruments).

Cell growth under treatments using Incucyte

In all experiments, 3,000 cells per well were seeded on a low-attachment 96-well plate (Corning, 3474). Cells were treated with the indicated concentrations of TMZ, Selinexor, importazole or irradiation (5Gy). Real-time growth was recorded using an Incucyte imaging system (Sartorius), with one image every 3 hours at 4X magnification using phase contrast and an RFP channel for 4 days. Incucyte software (Sartorius) was used for image analysis. Segmentation was performed on red channel using Top-Hat method with a radius of 100um and a threshold of 1 RCU. Cell growth was quantified as RFP channel object counts per well.

Comet assay

37°C molten LM agarose and cell suspension ($1.5x10^5$ cells/ml) were prepared in a 10:1 (v/v) ratio. 50uL was poured onto the comet slide (Trevigen Comet assay kit, #4250-050-K), and immediately place at 4°C in the dark for 10min in a low humidity dust-free environment, to maximize agarose adherence on slides. Slides were irradiated with a dose of 5 Gy (rate of 1 Gy/min) using a gamma-ray GSR D1 irradiator Cs-137 (Gamma-Service Medical GmbH). Slides were then placed in an ice-cold lysis solution for 60 min at 4°C, followed by a freshly prepared alkaline unwinding solution bath (pH>13: 200 mM NaOH, 1 mM EDTA in 50 ml of dH2O) for 1 h at 4°C in the dark. Next, the slides were placed in an electrophoresis chamber filled with alkaline buffer (200 mM NaOH, 1 mM EDTA in 1 L dH2O). Electrophoresis was performed at 28 V for 30 min. Slides were recovered and excess solution removed, rinsed in

dH2O and then in 70% ethanol for 5 min each. Slides were dried in a 37°C incubator for 10-15 min and stored with a desiccant prior to scoring. Cells were visualized by SYBR green staining, and 200 randomly-selected cells per slide were analyzed (various parameters including mean olive moment) under a light microscope using the Comet Assay Metafer software from Metasystems.

Transmission Electron Microscopy

For transmission electron microscopy (TEM), pellets of the sorted populations (hybrid, hybrid-low, bulk) were mixed with a fixative containing 2.5% glutaraldehyde and 4% paraformaldehyde in 0.1 M sodium cacodylate buffer (pH 7.4) at room temperature for 1.5 h. Cells were rinsed with 0.1 M sodium cacodylate buffer (pH 7.4) and then postfixed in 1% osmium tetroxide in 0.1 M sodium cacodylate buffer (pH 7.4) at room temperature for 1 h. The cells were dehydrated using an ethanol series, which was then replaced with acetone. The cells were infiltrated with a mixture of Epon resin diluted in acetone and ultimately embedded in absolute resin.

Ultrathin sections were cut on a Leica EM UCT ultramicrotome and double stained with Uranlyless and lead citrate (EM Grade, France). The ultrathin sections were observed using a JEOL JEM-2200FS transmission electron microscope.

Animals

Tumor growth monitoring and treatment protocols. Orthotopic implantation of the patient-derived MGG4 cell line, tumor growth monitoring and treatment of mice were performed as described[26]. Briefly, 100.000 MGG4-Gluc cells in 5 µl of media were implanted and tumor growth was routinely monitored using the Gluc assay in the bloodstream, until reaching the predefined threshold of 3 million RLU (Relative Luminescence Units) on average 90 days post implantation. In vivo IR was performed using an image-guided micro X-ray with 10 Gy per mouse. For TMZ, 10 mg/kg was administered intraperitoneally. 7 days post-treatment, the mice were euthanized for tumor harvesting and RNA sequencing. RNA-Seq was aligned with the human genome to account for MGG4 GBM cells only, thus avoiding the involvement of tumor microenvironment.

Survival experiment. For Figure 6F, MGG8 cells were sorted based on fluorescence intensity to obtain bulk, hybrid and hybrid-low populations. For Figure 7H, MGG8 cells were stably transduced with shKPNA2 or shCTRL and the silencing efficiency was tested by qPCR. 20,000 cells in 5 µl of media were implanted orthotopically as described[26]. Mice behaviour and health were checked routinely for signs of neurological deficits, lethargy, weight loss, ataxia or seizures. Mice were euthanized if they reached the limit points as defined by ethical

committees (based on body condition, neurological assessment, and tumor burden evaluation by GLuc bioluminescence) to minimize animal suffering and ensure compliance with ethical guidelines. To exclude mice where the tumor implantation failed, we excluded mice with a survival longer than 60 days for Figure 6F and longer than 38 days for Figure 7H.

Single cell Chromatin Immunoprecipitation

The full single-cell ChIP-seq protocol was previously described[24]. Briefly, 1M cells were incubated for 20 min in 1 µM CellTrace solution (CellTrace CFSE, Thermo Scientific, C34554) at room temperature. Cells were then washed and incubated in culture medium for 15 min to allow the CellTrace reagent to undergo acetate hydrolysis. Labeled cells were resuspended in a suspension mix (PBS supplemented with 30% Percoll, 0.1% Pluronic F-68, 25 mM HEPES pH 7.4 and 50 mM NaCl) and encapsulated in 45pL droplets with a digestion mix (0,1 M Tris-HCl pH 7.4, 0,3 M NaCl, 2,15% Tritonx100, 0,22% NaDoc, 0,01 M $CaCl_2$, 0,2 U/µl Micrococcale Nuclease (Thermo Scientific, EN0181), 1x Protease Inhibitor Cocktail and 2µM Sulforhodamine B). Once encapsulated, cells droplets are collected and incubated at 37°C for 20 minutes for cellular lysis and chromatin digestion.

In parallel, beads with unique double-stranded barcodes were encapsulated in 100pL droplets with a ligation mix (0,1 M EGTA, 2 mM ATP, 2 µM Sulforhodamine B, 0,38 U/µL Fast link DNA ligase (Lucigen, LK0750H)) and a repair mix (4 mM dNTP, 2 µM Sulforhodamine B, 0,1 x End-it Repair mix (Lucigen, ER81050), 0,1 U/µL Fast link DNA ligase (Lucigen, LK0750H)).

Bead droplets and cell droplets were then fused in a 1:1 ratio by electrocoalescence and counted by laser detection, as previously described[24]. Double-stranded DNA barcodes were released into fused droplets by UV exposure (1 min, 230mW/cm^2) and ligated to nucleosomes during an overnight incubation at 16°C.

Immunoprecipitation of barcoded nucleosomes associated with the H3K27me3 histone mark (Antibody Cell Signaling Technology, 9733-C36B11, 1/200) or H3K4me3 histone mark (Antibody Cell Signaling Technology, 9751-C42D8, 1/200) was performed overnight at 4°C on a rotating wheel. DNA amplification and library preparation were performed as described[24]. Libraries were then sequenced on NovaSeq 6000 in PE100, with a coverage of 100,000 reads per cell.

scChIP-seq analysis

scChIPseq data were processed using the pipeline scChIP-inDrop v1.1.0 (https://doi.org/10.5281/zenodo.10523263)[24, 56]. Briefly, reads were aligned to the hg38 reference genome using STAR (v2.7.8a), and PCR and RT duplicates were removed to produce 50kb binned count matrices. The scChIP-seq data were analyzed in R using the Signac and Seurat packages. The thresholds were set to each feature expressed in a minimum

of ten cells and each cell expressed at least thousand features. The top one percent cells expressing the most features were removed to avoid contamination with gDNA. Rainer J (2017). Each feature was annotated according to the human genome GRCh38-hg38 using EnsDb.Hsapiens.v86 R package (EnsDb.Hsapiens.v86: Ensembl-based annotation package. R package version 2.99.0.). The data were normalized by the term frequency-inverse document frequency (TF-IDF) with a scale factor of 10,000, and the dimensions were reduced by the singular value decomposition (SVD) method. Nonlinear dimension reduction was applied using the Uniform Manifold Approximation and Projection (UMAP) method on dimensions 2 to 20. Clustering was performed using a smart local moving (SLM) algorithm with a resolution of 0.7. Trajectories were built from the UMAP using monocle3. UMAP and trajectory plots were generated using R, Seurat, and Monocle3. Peak calling was performed using the European Public Galaxy Server (www.usegalaxy.eu). The reads were aligned to the human genome GRCh38-hg38 using Bowtie2. Peaks were called using MACS2 with an FDR threshold of 1e-05. The peaks were annotated using the ChIPseeker. Unpoised genes were defined as genes with an H3K4me3 peak in their promoter and no H3K27me3 peak in their promoter/gene body. Enrichment analyses were performed using the EnrichR software (https://maayanlab.cloud/Enrichr/). Motif enrichment analysis was performed using MEME suite (https://meme-suite.org/meme/).

RNA sequencing

Total RNA was extracted using the QIAGEN RNeasy Mini kit (#74104) according to the manufacturer's instructions, including a DNase digestion step. RNA concentration and quality were determined using Nanodrop (ThermoFisher). RNA-seq libraries were built using the Illumina library structure (Illumina) and sequenced as 100bp paired-end runs on Novaseq 6000 (Illumina). An average of 30 million reads per sample were sequenced.

The resulting reads were processed using the Institut Curie Nextflow pipeline v3.1.8 (in-vivo MGG4, MGG4 TMZ), v2.1.1 (MGG4-MGG8-P3 irradiated), v4.0.1 (MGG8 and MGG4 hybrid). The quality of reads was assessed using FastQC, mapped to the reference genome (hg19/GRCh37 or hg38/GRCh38 for hybrid cells) using the STAR software, and raw read count tables were generated using STAR. Versions of the software and full pipelines are available at https://github.com/bioinfo-pf-curie/RNA-seq/.

Differential expression analysis was performed using the DESeq2 R package v1.30.0. DESeq2 median ratio normalization was extracted to be used as an input for Gene Set Enrichment Analysis (GSEA), performed using the Broad Institute software. Publicly available gene pathway databases were used for GSEA analyses (HALLMARK, KEGG, and Gene Ontology), along with custom-made signatures. Over-representation analysis (ORA) was performed using the DecoPath webserver tool (https://decopath.scai.fraunhofer.de/) and the Reactome website

(https://reactome.org/). Ingenuity Pathways Analysis (IPA) was performed using the Qiagen platform. Both Reactome and IPA are based on manually curated and updated datasets. RNA-seq data were deposited in NCBI's Gene Expression Omnibus (GEO) and are accessible through GSE256067.

Spatial transcriptomics of GBM patients

Spatial transcriptomic RNA data was obtained from a previously published data repository[57]. Briefly, data were analyzed and quality controlled using the cell ranger pipeline provided by 10X Genomics. The data were imported to create the Seurat object. Gene expression was normalized and scaled using a regression model that included a sample batch and the percentage of ribosomal and mitochondrial gene expression. For spatial expression plots, we used enrichment scores of defined genesets generated using the “addmodulescore” function and plotted using “spatialfeatureplot”. Spatial correlation analysis was carried out over all samples in the dataset and plotted using the ggcorrplot packages implemented in R (Kassambara A (2023). _ggcorrplot: Visualization of a Correlation Matrix using 'ggplot2'_. R package version 0.1.4.1, <https://CRAN.R-project.org/package=ggcorrplot>).

Single cell RNA sequencing and analysis

Cell preparation. Cells were dissociated as described, washed and dead cells were removed using the dead cell removal kit (Miltenyi Biotech, #130-090-101). Briefly, the cells were incubated for 15 min at room temperature with dead cell removal microbeads, diluted in 1X binding buffer, and loaded into the column. The effluent contained live cells, and the column was washed with 1X binding buffer to maximize efficiency. Live cells were centrifuged, resuspended in 1X PBS BSA 0.04%, counted and resuspended at 10^6 cells/ml.

10X genomic procedure. The Chromium Single Cell 3’ v3.1 Library and Gel Bead kit (PN-1000121) and Chromium Next GEM Chip G Single Cell Kit (PN-1000127) were used according to the manufacturer’s instructions. 3000 cells were loaded on the chip, then 10X capture and library protocol was used without modification and samples were sequenced on the Illumina Novaseq 6000.

Data preprocessing. Output reads were converted to FASTQ files using *bcl2fastq* (v2.20), aligned to the hg38 reference genome with *Kalisto* (v0.46.2), and corrected bus files were used to generate raw count matrix using *bustools* (v.0.40.0). Filtering of the raw matrix for empty droplets was performed using the *cellranger* method in the *DropletUtils* package.

The filtered counts were imported into the Seurat R package for further processing. Raw transcript counts were filtered to remove cells with a total UMI lower than 2000 and higher than 7000, along with cells with more than 25% of mitochondrial genes. Normalization of count

matrices was performed using the *SCTransform* method (vst2 flavor version) and cells were regressed based on cell cycle scores.

Dimension reduction. Linear dimension reduction was performed using the 3000 genes with the highest variance as identified by SCTransform and 35 principal components, chosen through Seurat's PCHeatmap and Elbowplot. Seurat uniform manifold approximation and projection (UMAP) was applied to the reduced data for visualization in a 2D space. The number of clusters was determined using the clustree package for optimal-resolution clustering.

MGG4 cells were prepared, sequenced, and processed as described[26].

Proteome

Sample preparation. MGG8 subpopulations were sorted as previously described. Proteins from the cytoplasmic and nuclear fractions were extracted using the Qproteome Cell Compartment Kit (Qiagen, 37502). Protein concentration was quantified using BCA assay (Thermofisher, 23225). 10µg of proteins were precipitated overnight at –20°C with 0.1 mol/L ammonium acetate glacial in 80% methanol (buffer 1). After centrifugation at 14,000 g and 4°C for 15 min, the resulting pellets were washed twice with 100 µL of buffer 1 and further dried under vacuum (Savant Centrifuge SpeedVac concentrator, Thermo Fisher Scientific). Proteins were then reduced by incubation with 10 µL of 5 mmol/L DTT at 57°C for 1 h and alkylated with 4 µL of 55 mmol/L iodoacetamide for 30 min at room temperature in the dark. Trypsin/LysC (Promega) was added at a 1:50 (wt/wt) enzyme/substrate ratio at 37°C overnight. The samples were then loaded onto a custom-made C18 StageTip for desalting. Peptides were eluted using 40:60 MeCN/H_2O in 0.1% formic acid and vacuum concentrated to dryness before reconstitution in 10 µL injection buffer containing 0.3% trifluoroacetic acid (TFA) and then analyzed by liquid chromatography-tandem mass spectrometry (LC-MS/MS).

LC-MS/MS Analysis. Online chromatography was performed using an RSLCnano system (Ultimate 3000, Thermo Scientific) coupled with an Orbitrap Eclipse mass spectrometer (Thermo Scientific). The peptides were trapped on a 2 cm Nanoviper Precolumn (i.d. 75 µm, C18 Acclaim PepMapTM 100, Thermo Scientific) at a flow rate of 3.0 µL/min in buffer A (2/98 MeCN/H2O in 0.1% formic acid) for 4 min to desalt and concentrate the samples. Separation was performed on a 110 cm nanoviper column (110 cm µPACTM Neo HPLC column, Thermo Scientific) regulated to a temperature of 50°C with a linear gradient from 2% to 32% buffer B (100% MeCN in 0.1% formic acid) at a flow rate of 750nL/min for the first 6 min and then at 300nL/min over 204 min. MS1 data were collected in an Orbitrap (120,000 resolution; maximum injection time 60 ms; AGC 4 x 105). Charge states between 2 and 4 were required for MS2 analysis, and a 30 s dynamic exclusion window was used. MS2 scans were performed in the ion trap in rapid mode with HCD fragmentation (isolation window 1.2 Da; NCE 30%; maximum injection time 35 ms; AGC 104)

Data Analysis. For identification, the data were searched against the *Homo Sapiens* (UP000005640_9606) UniProt database using Sequest HT in Proteome Discoverer (version 2.4). Enzyme specificity was set to trypsin, and a maximum of two missed cleavage sites was allowed. Oxidized methionine, N-terminal acetylation, methionine loss, and methionine acetylation loss were set as the variable modifications. The maximum allowed mass deviation was set to 10 ppm for the monoisotopic precursor ions and 0.6 Da for MS/MS. The resulting files were further processed using myProMS[58] (https://github.com/bioinfo-pf-curie/myproms) v.3.10.0. False-discovery rate (FDR) was calculated using Percolator[59] and set to 1% at the peptide level for the entire study. Label-free quantification was performed using peptide extracted ion chromatograms (XICs) computed with MassChroQ[60] v.2.2.1. For protein quantification, XICs from proteotypic peptides shared between replicates (TopN matching) with missed cleavage were used. Median and scale normalization at the peptide level were applied to the total signal to correct the XICs for each biological replicate (N=3). To estimate the significance of the change in protein abundance, a linear model (adjusted for peptides and biological replicates) was used, and p-values were adjusted using the Benjamini–Hochberg FDR procedure. Proteins with at least two total peptides in all replicates (n=3), a 1.2-fold enrichment, and an adjusted p-value ≤ 0.05, were considered significantly enriched in sample comparisons. Proteins selected using these criteria were further analyzed (GO functional enrichment analysis and EnrichR pathway analysis). The mass spectrometry proteomics data have been deposited in the ProteomeXchange Consortium (http://proteomecentral.proteomexchange.org) via the PRIDE partner repository[61] with the dataset identifier PXD050217 (username: reviewer_pxd050217@ebi.ac.uk , password: SdH6MJ92)

Data mining for TMZ non-responders GBM cell lines

Viability analysis for sample classification. Data was obtained from the corresponding author upon request. The samples were classified as responsive or not to TMZ challenge. Cell cultures that exhibited viability below 70% post-challenge were classified as responders. A statistical comparison between the two groups revealed a significant reduction in viability in the responder group.

RNAseq analysis. Count matrices were downloaded from GEO (GSE232173) and the samples were grouped based on their viability, as described above. The count matrix was processed to remove genes with expression lower than 5 counts, leaving us with 19879 genes across 19 samples. This matrix was used to construct a DEseq object[62], with the TMZ response as a differentiating factor. Differential sequencing analysis revealed 108 differentially expressed genes (padj <0.05).

GSVA analysis._Gene Set Variation Analysis (GSVA), a non-parametric, unsupervised method that estimates the variation of gene set enrichment through the samples of a dataset. GSVA allows the evaluation of predetermined genesets. The enrichment score of each gene set was determined by ranking the genes based on their expression levels in each sample. Subsequently, for each geneset, the method evaluates the positions of the genes in the ranked list and calculates an enrichment score based on the empirical cumulative distribution function. The resulting geneset enrichment scores were then used to determine the expression of the hybrid geneset, wrt to TMZ response status. We used the GSVA implementation in R[63].

Publicly-available datasets

Feature plot of the hybrid signature on the cell hierarchy transcriptome map by Neftel was created using the web-based platform from The Broad Institute (https://singlecell.broadinstitute.org/single_cell).

MES versus PN differentially expressed genes (log2FC>0.5; pvalue<0.001) were used as input on the web platform to obtain the average expression per cell, subdivided in mesenchymal cells (MES and AC-like cells) and proneural cells (OPC- and NPC-like cells). Plots were generated on R using ggplot2 (v3.5.2) package.

For the GLASS Consortium analyses, data was downloaded from https://glass-consortium.org. GSEA of log FC between the first recurrence and the primary sample was performed in patients with GBM. The inclusion criteria for patients were as follows: histology=Glioblastoma; idh_status=IDHwt; surgery number=1+2; second surgery with IR+TMZ or no treatment.

For deconvolution, 19,531 tumor cells from a publicly available scRNA-seq dataset[28] of 5 IDH1-wildtype gliomas were automatically annotated with SingleR (v3.19) using the transcriptomic profiles of MGG8 and MGG4 (hybrid cells) as a reference. Subsequently, the entire IDH-wildtype dataset (including tumor cells and microenvironment) was used as a reference for deconvolution of 292 previously published bulk RNA-seq samples from IDH-wildtype glioma[10]. A signature matrix was created using the 'Create Signature Matrix from the scRNA-seq reference module of CIBERSORTx[64] with default parameters, replicates = 10, and quantile normalization disabled. The reference was downsampled to 5,000 cells using the 'sample' command in R and with the seed set to 11 to meet platform recommendations. Cell fractions were then estimated using the CIBERSORTx webserver (https://cibersortx.stanford.edu/) in relative mode, with quantile normalization and batch correction disabled (default settings), and 100 permutations for significance analysis.

For all 118 primary tumors in the GLASS cohort having overlapping RNA and DNA sequencing data available, genetic alterations were gathered from https://www.synapse.org/glass. The association between the most prevalent CNVs or mutations in GBM and the deconvoluted percentage of hybrid cells among total tumor cells was then tested using a Wilcoxon test.

For the IvyGAP analysis, data was downloaded from https://glioblastoma.alleninstitute.org/. GSEA was performed between the core tumor samples and the rest of the tumor regions (infiltrating tumor, leading edge, microvascular proliferation, and pseudopalisading cells around necrosis).

For Supplementary Figure 4, scRNA-seq analysis was performed using a publicly available integrated and harmonized dataset from the GBM microenvironment (doi:10.5281/zenodo.6962901). This dataset is composed of 16 integrated datasets, with 338,564 cells from a total of 110 patients. The dataset comprised 127,521 neoplastic cells and 211043 cells from the local microenvironment, all annotated at multiple levels. The data were analyzed and visualizations were created using the Seurat package for scRNA-seq data. Dotplots of gene expression across all cellular populations was carried out using the “Dotplot” function implemented within Seurat. All analyses were performed using the R computing environment.

Correlations between Myc targets and hybrid cells in the GBM cell lines were performed using the DepMap portal (https://depmap.org/portal/).
